# Bispecific aptamer-decorated and light-triggered nanoparticles targeting tumor and stromal cells in breast cancer derived organoids: implications for precision phototherapies

**DOI:** 10.1186/s13046-024-03014-x

**Published:** 2024-03-26

**Authors:** Simona Camorani, Alessandra Caliendo, Elena Morrone, Lisa Agnello, Matteo Martini, Monica Cantile, Margherita Cerrone, Antonella Zannetti, Massimo La Deda, Monica Fedele, Loredana Ricciardi, Laura Cerchia

**Affiliations:** 1 Institute of Experimental Endocrinology and Oncology “Gaetano Salvatore”, National Research Council, 80131 Naples, Italy; 2 CNR-NANOTEC Institute of Nanotechnology, National Research Council, Rende, CS Italy; 3 Department of Chemistry and Chemical Technologies, University of Calabria, Rende, CS Italy; 4 Department of Chemistry, Biology and Biotechnology, University of Perugia, Perugia, Italy; 5 Institute of Light and Matter, UMR 5306, Claude Bernard University Lyon 1, Villeurbanne, France; 6 Institutional Biobank-Scientific Directorate, National Cancer Institute INT-IRCCS Fondazione G. Pascale, 80131 Naples, Italy; 7 Pathology Unit, National Cancer Institute INT-IRCCS Fondazione G. Pascale, 80131 Naples, Italy; 8 Institute of Biostructures and Bioimaging, National Research Council, 80145 Naples, Italy

**Keywords:** Aptamer, EGFR, PDGFRβ, Dual targeting, Nanomedicine, Patient-derived cancer organoids, Phototherapy, Tumor microenvironment

## Abstract

**Background:**

Based on the established role of cancer-stroma cross-talk in tumor growth, progression and chemoresistance, targeting interactions between tumor cells and their stroma provides new therapeutic approaches. Dual-targeted nanotherapeutics selectively acting on both tumor and stromal cells may overcome the limits of tumor cell-targeting single-ligand nanomedicine due to the complexity of the tumor microenvironment.

**Methods:**

Gold-core/silica-shell nanoparticles embedding a water-soluble iridium(III) complex as photosensitizer and luminescent probe (Ir_en_-AuSiO_2__COOH) were efficiently decorated with amino-terminated EGFR (CL4) and PDGFRβ (Gint4.T) aptamers (Ir_en_-AuSiO_2__Aptamer). The targeting specificity, and the synergistic photodynamic and photothermal effects of either single- and dual-aptamer-decorated nanoparticles have been assessed by confocal microscopy and cell viability assays, respectively, on different human cell types including mesenchymal subtype triple-negative breast cancer (MES-TNBC) MDA-MB-231 and BT-549 cell lines (both EGFR and PDGFRβ positive), luminal/HER2-positive breast cancer BT-474 and epidermoid carcinoma A431 cells (only EGFR positive) and adipose-derived mesenchymal stromal/stem cells (MSCs) (only PDGFRβ positive). Cells lacking expression of both receptors were used as negative controls. To take into account the tumor-stroma interplay, fluorescence imaging and cytotoxicity were evaluated in preclinical three-dimensional (3D) stroma-rich breast cancer models.

**Results:**

We show efficient capability of Ir_en_-AuSiO_2__Aptamer nanoplatforms to selectively enter into target cells, and kill them, through EGFR and/or PDGFRβ recognition. Importantly, by targeting EGFR^+^ tumor/PDGFRβ^+^ stromal cells in the entire tumor bulk, the dual-aptamer-engineered nanoparticles resulted more effective than unconjugated or single-aptamer-conjugated nanoparticles in either 3D spheroids cocultures of tumor cells and MSCs, and in breast cancer organoids derived from pathologically and molecularly well-characterized tumors.

**Conclusions:**

Our study proposes smart, novel and safe multifunctional nanoplatforms simultaneously addressing cancer-stroma within the tumor microenvironment, which are: (i) actively delivered to the targeted cells through highly specific aptamers; (ii) localized by means of their luminescence, and (iii) activated via minimally invasive light, launching efficient tumor death, thus providing innovative precision therapeutics. Given the unique features, the proposed dual targeted nanoformulations may open a new door to precision cancer treatment.

**Supplementary Information:**

The online version contains supplementary material available at 10.1186/s13046-024-03014-x.

## Background

Diverse components of the breast cancer microenvironment, including fibroblasts, mesenchymal stem cells, macrophages, adipocytes and altered extracellular matrix, synergistically promote tumor growth, invasion and metastasis, and resistance to therapy [1]. A continuous remodeling of the architecture of the tumor occurs in response to the dynamic signaling between tumor cells and stromal cells [2]. These stromal cells are actively recruited from the other tissues to the tumor site, where they shift from a neutral or anti-tumor behavior toward a pro-tumorigenic role [3]. Mesenchymal stem/stromal cells ″educated″ by tumor cells promote malignant features including proliferation, epithelial-mesenchymal transition, propagation of cancer stem cells, angiogenesis, inhibition of apoptosis, immune system suppression, evasion of immune surveillance and drug resistance [4]. Therefore, in the search for new effective anticancer therapies, it is mandatory to take into account the complex cross-talk between cancer cells and neighboring stromal cells. In this context, procedures for developing three-dimensional (3D) stroma-rich coculture models are increasing fast as they enable investigations related to intercellular dialogue within the tumor microenvironment (TME). Multicellular tumor spheroids, consisting of tumor and stromal cells, and patient-derived cancer organoids (PDCOs) recapitulating in vitro the complex structure and function of the original cancer, are more accessible than a living system in a variety of biological studies [5–7] and are essential for cancer research and drug development [8].

The rapid development of nanomaterials has led to remarkable advances in the field of cancer treatment [9]. Among them, light-responsive nanomaterials have received a great deal of attention for application in phototherapy, i.e. Photodynamic Therapy (PDT) and Photothermal Therapy (PTT) [10–12]. In PDT, photosensitizers absorb and transfer light energy to surrounding molecules, generating cytotoxic reactive oxygen species, resulting in the activation of apoptotic processes; in particular, nanomaterials are used in PDT as carriers or as active agents [13, 14]. In PTT, photothermal conversion agents are able to capture light energy and convert it into heat, triggering cancer cell death by temperature-dependent necrosis [15]. Among all, noble-metal-based nanoparticles, due to their thermoplasmonic properties, proved to be efficient nano-sources of heat for PTT application [16]. Both treatment approaches, as well as to the possibility of being used in combination to develop synergistic effects [15], ensure a high spatio-temporal control of the cytotoxic activity in the limited area exposed to irradiation, thus limiting systemic side effects. In this frame, we have previously reported the synthesis and characterization of a multifunctional nanoplatform for combined PDT and PTT treatments, based on a gold-core and silica-shell structure, embedding in the polysiloxane matrix an iridium(III) compound ([Ir(ppy)_2_(en)]OOCCH_3_, where ppy = 2-phenylpyridine and en = ethylenediamine, Ir_en_), employed as photosensitizer and luminescent probe [17, 18].

Here, we succeeded in preparing a multifunctional nanosystem having two different RNA aptamers conjugated on the external surface of Ir_en_-embedded gold-core/silica-shell-based nanoparticles for synergistic PDT and PTT on either 3D cocultures of tumor cells and stromal cells, and breast cancer derived organoids. Specifically, for tumor cell targeting we used the CL4 2′Fluoro-pyrimidines (2′F-Py) RNA aptamer (Kd, 10 nM; 39 nt) [19], capable of binding at high efficacy to the extracellular domain of epidermal growth factor receptor (EGFR), one of the most potent oncoprotein of human cancer. The aptamer has excellent capability to recognize EGFR-positive cells belonging to different cancer types [19, 20], including human epidermal growth factor receptor 2 (HER2)-positive tumors [21, 22] and triple-negative breast cancer (TNBC) [23]. Moreover, due to its selectivity, CL4 has been extensively used by our group [24] and others [25–29] to decorate different kinds of drug-loaded nanoformulations to target breast cancers implanted in mice.

On the other hand, for stromal cells targeting we used the 2′F-Py RNA Gint4.T aptamer (Kd, 9.6 nM; 33 nt) [20], which binds to the extracellular domain of platelet-derived growth factor receptor β (PDGFRβ), an established marker of stromal cells, including mesenchymal stem cells [30], cancer associated fibroblasts [31, 32], tumor-associated endothelial cells [33], immune cells [34, 35], and macrophages [36–38]. Our previous studies showed the ability of Gint4.T to bind to/inhibit PDGFRβ that is expressed on the surface of TNBC cells of the highly malignant and invasive mesenchymal subtype (MES) [39], accordingly to their undifferentiated and mesenchymal phenotype. Importantly, the aptamer binds to different TME components, including mesenchymal stem cells [30], T cells [35] and endothelial cells of vessels that vascularize the tumor [40], thus ultimately hampering their pro-tumorigenic function.

Our results show for the first time the striking potential of the dual EGFR and PDGFRβ aptamer-functionalized nanosystems for simultaneous targeting and photo-induced killing of breast tumor cells and stromal cells within the TME. The proposed strategy will represent a significant advance in nanomedicine, as it can be adapted to treat other tumors as well.

## Methods

### Chemicals and aptamers

4-(1,1,3,3-Tetramethylbutyl)phenyl-polyethylene glycol (Triton X-100), n-hexanol, cyclohexane, ammonium hydroxide solution (28% w/w), hydrogen tetrachloroaurate (III) trihydrate (HAuCl_4_·3H_2_O), sodium 2-mercaptoethanesulfonate (Mesna), sodium borohydride (NaBH_4_), (3-aminopropyl)triethoxysilane (APTES), tetraethoxysilane (TEOS), N-(3-dimethylaminopropyl)-N′-ethylcarbodiimide hydrochloride (EDC), N-hydroxysuccinimide (NHS) and phosphate buffer saline tablets were purchased from Sigma-Aldrich (Saint Louis, MO, USA). 11-Triethoxysilylundecanoic acid (95%) and N-(3-triethoxysilyl) propylsuccinic anhydride (94%) were purchased from ABCR (Karlsruhe, Germany). Ultrapure water (Milli-Q, 18 MΩ·cm) was used for the preparation of the aqueous solutions and for all rinses. Phosphate buffer solution (PBS, pH 7.4) was prepared by dissolving one phosphate buffer saline table in 200 mL of Milli-Q water. All other solvents used were of analytical grade.

NH_2_-terminated 2′F-Py-containing RNA, CL4, its scrambled sequence (Scr) used as a negative control, and Gint4.T were synthesized by LGC Biosearch Technologies (Risskov-Denmark).

The sequences are as follows:

- CL4: 5' (NH_2_-C6)GCCUUAGUAACGUGCUUUGAUGUCGAUUCGACAGGAGGC 3'
- Scr: 5' (NH_2_-C6)UUCGUACCGGGUAGGUUGGCUUGCACAUAGAACGUGUCA 3'.
- Gint4.T: 5' (NH_2_-C6)UGUCGUGGGGCAUCGAGUAAAUGCAAUUCGACA 3'.

Before usage, aptamers were heated at 85 °C for 5 min, snap-cooled on ice for 3 min, and allowed to warm up for 10 min to 37 °C, before incubation with the cells for checking appropriate folding, or to room temperature (RT) for nanoparticles conjugation.

### Synthesis of nanoparticles

Multifunctional gold-core/silica-shell nanoparticles embedding the photosensitizing and luminescent molecule Ir_en_ [41] were synthesized following a previously reported protocol [17, 18] with slight modifications. Briefly, quaternary water/oil (W/O) microemulsion was prepared by mixing 3.6 mL of Triton X-100, 3.6 mL of n-hexanol, 15 mL of cyclohexane and a water solution consisting of 0.9 mL HAuCl_4_·3H_2_O (12.75 mM), 0.9 mL Mesna (36.5 mM) and 0.3 mL NaBH_4_ (423 mM). Then, Ir_en_ (7 mg/0.1 mL water) was added to the microemulsion, followed by the addition of 0.010 mL of APTES and 0.150 mL of TEOS. After 30 min, 0.080 mL of ammonium hydroxide solution was added. The mixture was stirred overnight at RT. Afterward, the functionalization of the nanoparticle surface with carboxyl-terminated aliphatic chains, was carried out by addition after 24, 24 + 3 and 48 h of 0.015 mL of 11-Triethoxysilylundecanoic acid. Finally, in order to improve the colloidal stability, the silane coupling agent N-(3-triethoxysilyl) propylsuccinic anhydride (0.015 mL) was added after 48 + 3 h. The mixture was stirred overnight at RT. Then, the microemulsion was broken by addition of isopropanol and water in a volume ratio 1:1:1. Purification steps by centrifugal ultrafiltration (Vivaspin 20 PES, 100,000 MWCO, Sartorius, Gottingen, Germany) allowed the complete removal of all unreacted species. The obtained nanoparticles (Ir_en_-AuSiO_2__COOH) were finally dispersed in water to a final volume of 20 mL and filtered by a 200 nm nylon membrane (Sartorius).

Ir_en_-AuSiO_2__COOH were conjugated with the selected aptamers through a covalent bond between the carboxyl group (− COOH) of the nanoparticle surface coating agent and the amino group (− NH_2_) on the 5′-end of RNA scaffold. 1 mL of EDC (26 mM) was mixed under stirring to 1 mL of Ir_en_-AuSiO_2__COOH nanoparticles solution. Then, 1 mL of NHS (24.3 mM) was added to the reaction solution. After 50 min, 3 mL of PBS were added, followed by the addition of 1 mL of an aqueous solution of CL4 (0.280 μM), Scr (0.280 μM) and Gint4.T (0.280 μM), respectively, and of 0.5 mL of CL4 (0.280 μM) and 0.5 mL of Gint4.T (0.280 μM) to get the dual aptamer-decorated preparation. The reaction continued for 24 h. Afterward, aptamer-nanoconjugates Ir_en_-AuSiO_2__CL4, Ir_en_-AuSiO_2__Scr, Ir_en_-AuSiO_2__Gint4.T and Ir_en_-AuSiO_2__CL4_Gint4.T were washed by centrifugal filter devices (Vivaspin 20 PES, 100,000 MWCO, Sartorius) to eliminate unconjugated aptamers and unbound reaction components, and concentrated to a final volume of 1 mL, then stored at 4 °C until use. The same procedure, without aptamers addition, was followed to obtain Ir_en_-AuSiO_2__COOH/NHS nanoparticles in order to use them as control.

### Characterization of nanoparticles

The synthesized Ir_en_-AuSiO_2__COOH nanoparticles were characterized by Transmission Electron Microscopy (TEM), Dynamic Light Scattering (DLS) and UV–Vis spectroscopy.

The morphology was observed using a JEOL 2010F transmission electron microscope. The sample was prepared by depositing a drop of a diluted colloidal solution on 200 mesh carbon-coated copper grids. After evaporation of the solvent in air at RT, the nanoparticles were observed at an operating voltage of 80 kV. The hydrodynamic sizes and ζ-potential values were measured with a Zetasizer Nano ZS (Malvern) instrument (632.8 nm, 4 mW HeNe gas laser, avalanche photodiode detector, 173° detection), using glass cuvettes (1 × 1 cm) and disposable folded capillary zeta cells, respectively, and the results expressed as average of three measurements. Extinction and excitation/emission spectra were recorded with a PerkinElmer Lambda 900 spectrophotometer and Perkin-Elmer LS-50B spectrofluorometer, using quartz cuvettes with a light path 1 × 0.4 cm. The calculation of the nanoparticles concentration (number of nanoparticles per mL) and the yield of encapsulation of Ir_en_ (number of Ir_en_ molecules per nanoparticle) were carried out according to the procedure previously reported [42]. Absorption spectra were acquired over time (over one month) to monitor the stability of the nanostructures in the aqueous medium.

To validate the presence of the target-specific aptamers on the nanoparticles surface, Ir_en_-AuSiO_2__CL4, Ir_en_-AuSiO_2__Scr, Ir_en_-AuSiO_2__Gint4.T and Ir_en_-AuSiO_2__CL4_Gint4.T were characterized by DLS and UV–Vis spectroscopy techniques.

To determine the amount of aptamer conjugated to the nanoparticles, reverse transcription-quantitative polymerase chain reaction (RT-qPCR) based assay was performed as previously described [40]. Briefly, Ir_en_-AuSiO_2__CL4, Ir_en_-AuSiO_2__Scr, Ir_en_-AuSiO_2__Gint4.T and Ir_en_-AuSiO_2__CL4_Gint4.T were incubated with aptamer specific 3′ primers, heated at 65 °C for 5 min and annealed at 22 °C for 5 min. RNA was reverse transcribed using Tetro Reverse Transcriptase (Bioline London, UK) at 42 °C for 15 min followed by an extension at 50 °C for 30 min and enzyme inactivation at 85 °C for 5 min. The products from the reverse transcription reaction were subjected to qPCR amplification. The sequences of aptamer-specific 5′ and 3′ primers for Gint4.T and Scr, and CL4 were reported in [40, 43], respectively. The conjugation efficiency was calculated as pmoles of aptamer_conjugated_/ aptamer_total_ (%).

### Cell lines and two-dimensional (2D) culture conditions

Human MES-TNBC MDA-MB-231 and BT-549, luminal B/HER2-positive breast cancer BT-474, luminal A/estrogen receptor and progesterone receptor-positive breast cancer MCF7, and epidermoid carcinoma A431 cell lines were purchased from the American Type Culture Collection (ATCC, Manassas, VA) and grown as previously reported [44]. Green fluorescent protein (GFP)-labeled BT-549 cells (BT-549-GFP) was produced as previously described [30] and grown in Roswell Park Memorial Institute-1640 medium (Sigma-Aldrich, Milan, Italy) supplemented with 10% fetal bovine serum (Sigma-Aldrich). Human adipose MSCs were purchased from Sigma-Aldrich (SCC038) and grown in Human Mesenchymal-XF Expansion Medium (Sigma-Aldrich) in a humidified incubator in 5% CO2 at 37 °C.

### Establishment of 3D spheroids of stromal cells and breast cancer cells

For 3D heterotypic tumor spheroids, 2 × 10^3^ cancer cells were mixed with 8 × 10^3^ MSCs (ratio 1:4) and seeded in 24-ultralow attachment plates (Corning Incorporate, Corning, NY) in Dulbecco's Modified Eagle Medium (DMEM)/F-12 medium (D8437 Sigma-Aldrich), supplemented with 2% Matrigel Basement Membrane Matrix Growth Factor Reduced (Corning Incorporate), B27 (1:50, Gibco™ by Invitrogen, Carlsbad, CA), 20 ng/ml basic fibroblast growth factor (Sigma-Aldrich) and 10 ng/ml epidermal growth factor (Sigma-Aldrich). For homotypic cultures, 2 × 10^3^ cancer cells or 8 × 10^3^ MSCs were seeded alone. Spheroid formation was checked daily using a phase-contrast microscopy (Leica DMI3000 B apparatus), images were captured and the diameter of spheroids was measured.

### Establishment of patient-derived breast cancer organoids

Breast cancer samples from three patients who underwent surgery at the National Cancer Institute ″Fondazione Giovanni Pascale″ of Naples, Italy, were enrolled in this study. This study was approved by the ethics committee of INT Pascale (Prot. CEI/390/15) and all the patients provided written informed consent. The tissue samples were collected for histopathological diagnosis and an aliquot was stored in the Institutional Biobank (BBI). Immunohistochemical staining was done on biobank histological formalin-fixated and paraffin-embedded tissue samples slides (4 μm), as previously reported [39], by using primary antibodies against PDGFRβ (dilution 1:50, rabbit monoclonal antibody, clone 28E1, Cell Signaling Technology Inc., Danvers, MA), and EGFR (dilution 1:100, rabbit monoclonal antibody, clone D38B1, Cell Signaling Technology Inc.). Results were interpreted using a light microscope. Ten fields on each of two cores and at least > 500 cells were analyzed for each sample. Two pathologists independently evaluated the intensity, extent and subcellular distribution of the immunostaining. Receptor expression was interpreted as positive when membranous and/or cytoplasmic (PDGFRβ) or membranous (EGFR) staining was observed.

Organoid development methods followed a previous reported procedure study [45]. Briefly, fresh tumor specimens were collected and transported in working medium (WM), consisting of DMEM-F/12 medium (Sigma-Aldrich) supplemented with 1 × Antibiotic–Antimycotic (Gibco™ by Invitrogen) and 10 μg/mL gentamycin (Euroclone, Milan, Italy), minced mechanically and then digested in WM (10 mL/g of tissue) supplemented with 2 mg/mL Collagenase Type II (Gibco™ by Invitrogen), for 16 h at 37 °C under shaking. The digested tissue was filtered through a 100 μm cell strainers (Corning Incorporate) followed by a 40 μm cell strainer (Corning Incorporate) to separate organoids from single cells, and then tumor fragments retained by the cell strainer were washed in WM. The suspension containing tumor organoids was centrifuged 5 min at 500 × g and the organoid pellet was plated in 24-ultralow attachment plates (Corning Incorporate) in the culture medium previously reported [45]. After 24 h, the organoids were centrifuged again at 500 × g and finally resuspended in 90% Matrigel Basement Membrane Matrix Growth Factor Reduced (Corning) diluted in WM and 35 μL drops were allowed to solidify in the inverted 24-well plates for 20 min at 37 °C. Organoids-Matrigel drops were then covered with 500 μL of culture medium and transferred into incubator for culturing. The culture medium was replaced every 3–4 days and organoids were passaged every 2–3 weeks at a split ratio of 1:2–1:3 using mechanical dissociation by pipetting or enzymatic digestion using TripLE Express (Gibco™ by Invitrogen) for 5–15 min at 37 °C.

### Immunoblotting

Cell lysates’ preparation and immunoblot analyses were performed as previously reported [35]. The filters were probed with the indicated primary antibodies: anti-EGFR, anti-PDGFRβ, anti-vinculin and anti-α-tubulin (Cell Signaling Technology Inc.). The blots shown are representative of at least three independent experiments.

### Flow cytometry

PDCOs were mechanically and enzymatically disaggregated into a single-cell suspension and then incubated with anti-EGFR or anti-PDGFRβ (dilution 1:50, R&D system, Minneapolis, MN) primary antibody diluted in Dulbecco’s phosphate buffered saline (DPBS)/BlockAid™ blocking solution (Invitrogen), for 15 min at RT. After three washes with DPBS, cells were incubated with Alexa Fluor 488 Anti-Goat (Invitrogen), washed three times in DPBS, suspended in 500 μl DPBS and analyzed by flow cytometry (BD Accuri™ C6). Data analysis was performed using FlowJo software (version 10.0.7).

### Confocal Microscopy

To evaluate uptake of nanoparticles in 2D cell systems, MDA-MB-231, BT-549, BT-474, A431, MCF7 cells (5.0 × 10^4^ cells/well in 24-well) or MSCs (4.0 × 10^4^ cells/well in 24-well) were seeded on the coverslip and, after 24 h, were incubated for 30 or 60 min at 37 °C with Ir_en_-AuSiO_2__COOH/NHS or Ir_en_-AuSiO_2__Aptamer nanoparticles, diluted at 5 μM Ir_en_ concentration in culture medium with 0.1 mg/mL yeast tRNA and 0.1 mg/mL ultrapure™ salmon sperm DNA (Invitrogen), as nonspecific competitors. After three washes with DPBS, cells were fixed with 4% paraformaldehyde in DPBS for 30 min at RT, permeabilized with 0.5% Triton X-100/DPBS for 5 min, subjected to nuclear staining with the NucRed 647 (Invitrogen), following the provider indications, and mounted with glycerol/DPBS. Wheat Germ Agglutinin-Alexa Fluor 488 conjugate (WGA-488) was used to visualize BT-549 cell membrane.

To test the ability of Ir_en_-AuSiO_2__Aptamer nanoplatforms to enter 3D models, heterotypic spheroids composed of BT-549-GFP or BT-474 tumor cells mixed with MSCs, and PDCOs (~ 100–200 μm diameter) were collected, centrifuged at 500 × g for 5 min, suspended in 90% Matrigel and 15-μL drops were deposited in prewarmed 8-well Chamber Slide (1 drop/well, Ibidi GmbH, Gräfelfing, Germany). Upon completed gelation, 200 μL of culture medium was added to each well. After 24 h, spheroids or PDCOs were incubated for 24 h at 37 °C with Ir_en_-AuSiO_2__COOH/NHS or Ir_en_-AuSiO_2__Aptamer nanoparticles, diluted at 5 μM Ir_en_ concentration in culture medium with nonspecific competitors. After three washes with DPBS, they were fixed, permeabilized and stained with NucRed 647, as described above. For α-smooth muscle actin (α-SMA) and fibroblast activation protein (FAP) staining, spheroids, fixed and permeabilized as described above, were subjected to blocking in 3% bovine serum albumin (BSA)/DPBS for 20 min at RT, incubated with anti-α-SMA (1:100, D4K9N, Cell Signaling Technology Inc.) or anti-FAP (1:50, SS-13, Santa Cruz Biotechnology Inc.) primary antibody diluted in 3% BSA/DPBS for 1 h at 37 °C, washed three times in DPBS, and incubated with Alexa Fluor 568 anti-rabbit or Alexa Fluor 568 anti-mouse (Invitrogen), respectively. Finally, glycerol/DPBS was added to each well.

Samples were visualized by Zeiss LSM 700 META confocal microscopy and imaging was performed using the following excitation wavelengths: 405 nm (Ir_en_), 488 nm (GFP; WGA-488), 555 nm (α-SMA; FAP), 639 nm (NucRed 647).

### Photodynamic effect of nanoplatforms in 2D cell culture

MDA-MB-231, BT-549, A431, MCF7 and BT-474 (7.0 × 10^3^ cells/well) and MSCs (4.0 × 10^3^ cells/well) were plated in 96-well microplates (Corning Incorporate) and, after 16 h at 37 °C, were either left untreated or treated for 1 h with 5 μM free Ir_en_ or Ir_en_-loaded nanoformulations (Ir_en_-AuSiO_2__COOH/NHS, Ir_en_-AuSiO_2__CL4, Ir_en_-AuSiO_2__Gint4.T, Ir_en_-AuSiO_2__CL4_Gint4.T or Ir_en_-AuSiO_2__Scr), diluted in cell culture medium at 5 μM Ir_en_ concentration, in the presence of 0.1 mg/mL yeast tRNA and 0.1 mg/mL ultrapure™ salmon sperm DNA (Invitrogen), as nonspecific competitors. After two washes with DPBS, fresh medium was added to the plate and the cells were kept in the dark or exposed to 254 nm light irradiation (maximal irradiance 4 W m − 2) for 1 h. Cell viability was evaluated 24 h after PDT treatment by Thiazolyl Blue Tetrazolium Bromide (MTT, AppliChem GmbH, Darmstadt, Germany), according to the manufacturer’s protocol.

### Photodynamic effect of nanoplatforms in 3D models

Heterotypic spheroids grown in 24-ultralow attachment plates up to reach a diameter of approximately 150–180 µm, were left untreated or treated for 24 h with Ir_en_-AuSiO_2__CL4, Iren-AuSiO_2__Gint4.T, Ir_en_-AuSiO_2__CL4_Gint4.T or Ir_en_-AuSiO_2__Scr (5 µM Ir_en_ concentration), diluted in culture medium in the presence of nonspecific competitors (0.1 mg/mL yeast tRNA and 0.1 mg/mL ultrapure™ salmon sperm DNA, Invitrogen). After two washes with DPBS, fresh culture medium was added to the plates and spheroids, either untreated and treated with nanoparticles, were exposed to 254 nm light irradiation (maximal irradiance 4 W m − 2) for 1 h. After 24 h, spheroids with a diameter greater than 50 µm were counted in at least 5 fields per condition, to monitor spheroid destruction. For cell viability assays, heterotypic spheroids or PDCOs (~ 100–200 µm diameter) were transferred (20 μL drop *plus* 90% Matrigel) into black clear bottom 96-well plates (Corning Incorporate) that were filled with culture medium. After 24 h, spheroids or PDCOs were left untreated or treated with each nano-formulation and irradiated as reported above. Cell viability was assessed by CellTiter-Glo® 3D (ATP) luminescent assay (Promega BioSciences Inc., San Luis Obispo, CA), according to the manufacturer’s protocol, using TECAN Infinite 200Pro microplate reader.

### Statistical analysis

Statistical values were defined using GraphPad Prism version 6.00 by one-way analysis of variance (ANOVA) followed by Tukey’s multiple comparison test. A *p*-value < 0.05 was considered significant for all analyses.

## Results

### Design, synthesis and characterization of the multifunctional Ir_en_-AuSiO_2__Aptamer nanoplatforms

Multifunctional light-responsive nanoplatforms were implemented by incorporating an Iridium-based metal complex, Ir_en_, with photosensitizing and luminescent properties [41], within the polysiloxane matrix of gold-core/silica-shell nanoparticles (Ir_en_-AuSiO_2__COOH).

**Fig. 1:**
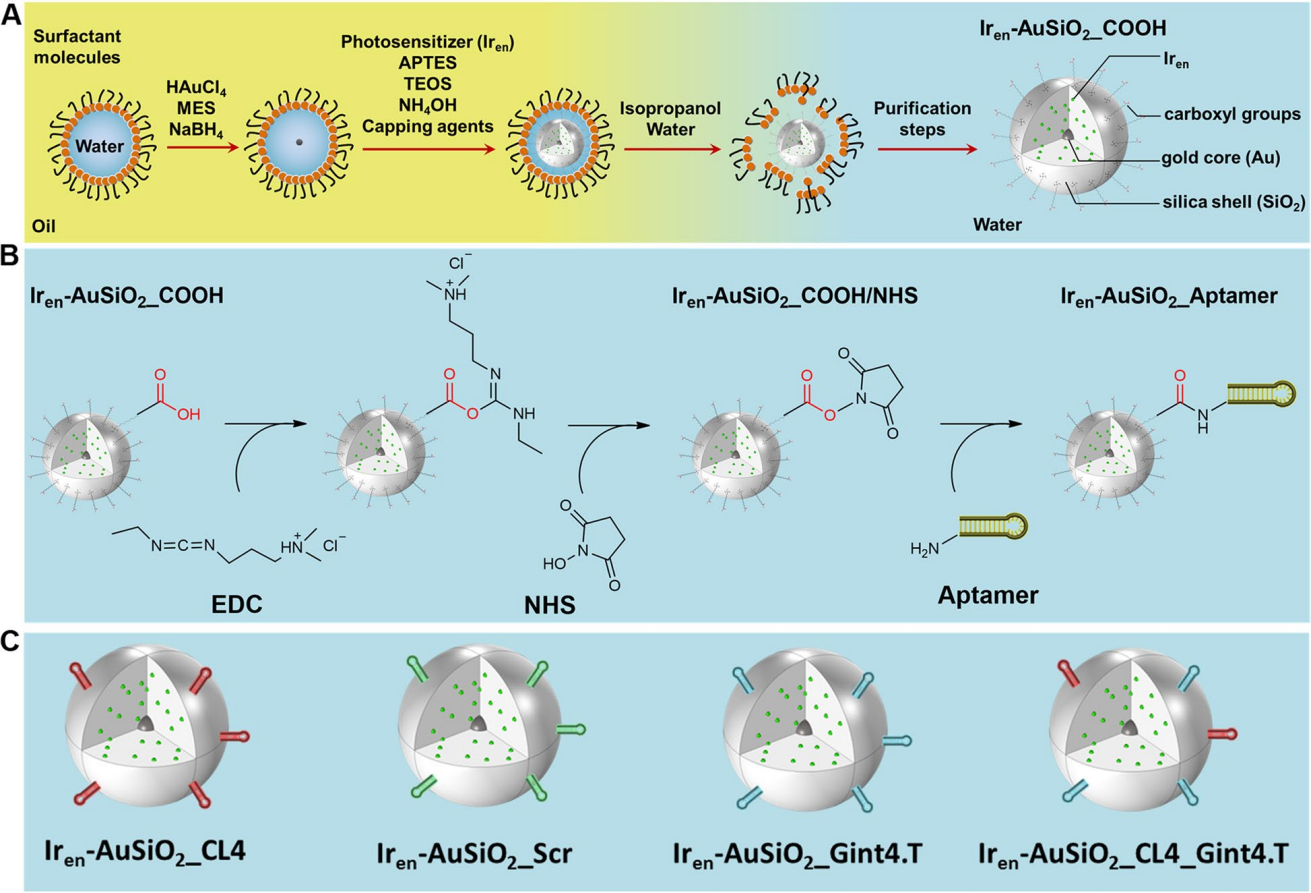
Schematic illustration of the protocol for the obtainment of the final nanoplatforms. **A** Ir_en_-AuSiO_2__COOH preparation by reverse microemulsion technique. Surfactant: 4-(1,1,3,3-Tetramethylbutyl)phenyl-polyethylene glycol; Mesna: sodium 2-mercaptoethanesulfonate; APTES: (3-aminopropyl)triethoxysilane; TEOS: tetraethoxysilane; capping agents: 11-Triethoxysilylundecanoic acid and N-(3-triethoxysilyl) propylsuccinic anhydride. **B** Covalent crosslinking strategy to achieve aptamers-nanoplatforms conjugates (Ir_en_-AuSiO_2__Aptamer). EDC: N-(3-dimethylaminopropyl)-N′-ethylcarbodiimide hydrochloride; NHS: N-Hydroxysuccinimide. **C** Ir_en_-AuSiO_2__Aptamer samples prepared and tested in this study

The preparation of Ir_en_-AuSiO_2__COOH was performed according to the well-known reverse microemulsion method [46] and a schematic representation of the synthetic procedure is illustrated in Fig. 1A. In a W/O microemulsion, water nanodroplets are stabilized by surfactant molecules and dispersed in a continuous oil phase. These reverse micelles act as nanoreactors, within which the homogenous and highly reproducible synthesis of nanoparticles takes place, minimizing the batch-to-batch variability. In the first step, the reduction of tetrachloroaurate(III) to Au^0^ leads to the formation of the gold core. Then, the addition of the silane precursors in alkaline environment gives rise, through hydrolysis and condensation processes, to the formation of the silica shell. The addition of the photosensitizing and luminescent Ir_en_ before the start of the polymerization process, ensures its physical incorporation into the polysiloxane matrix. Finally, the nanoparticles surface was functionalized with a coating agent containing a siloxy alkyl chain with a terminal carboxyl group. The full characterization of the Ir_en_-AuSiO_2__COOH nanoparticles, morphological features, surface charge, optical properties, photosensitizing and thermoplasmonic abilities, was reported in the Supplementary Information. TEM images revealed a homogeneous population of spherical gold-core/silica-shell particles, with an average size of 49.13 ± 4.28 nm and a gold core of 6.31 ± 0.81 nm (Supplementary Fig. 1A). Ir_en_-AuSiO_2__COOH are characterized by a hydrodynamic diameter of 85.51 ± 1.17 nm, Polydispersity Index (PDI) of 0.148 and a negative ζ-potential value of -23.5 ± 3.95 mV (Supplementary Fig. 1B). Similar ζ-potential values are reported for silica surfaces functionalized with terminal carboxyl groups in neutral aqueous medium [47, 48]. The successful loading of Ir_en_ within the nanoparticle silica shell was confirmed by UV–Vis spectroscopy (Supplementary Fig. 1C,D). In particular, the extinction spectrum of the colloidal solution of Ir_en_-AuSiO_2__COOH shows a broad band centered at 520 nm, corresponding to the characteristic Localized Surface Plasmon Resonance (LSPR) of the spherical gold core [49], and a more intense absorption band at lower wavelengths (250–300 nm), due to electronic transitions involving the Ir_en_ molecule [41]. Under light excitation, the emission spectrum of Ir_en_-AuSiO_2__COOH presents the characteristic band of the Iridium compound, peaked at 520 nm. The goodness of the emission spectrum was confirmed by the excitation one (Supplementary Fig. 1D).

In order to validate the photo-triggered properties of the obtained nanoplatforms, light-induced singlet oxygen generation and photothermal effects were assessed (see Supplementary Information for details). In particular, the singlet oxygen generation capability was evaluated by chemical method using 9,10-Anthracenediyl-bis(methylene)dimalonic acid (ABDA) as detection probe [50]. The photooxidation of ABDA in presence of Ir_en_-AuSiO_2__COOH was monitored by measuring its absorbance at 378 nm; while the absorbance attenuation of ABDA was negligible for the control solution (see Supplementary Information and Supplementary Fig. 2A), in presence of Ir_en_-AuSiO_2__COOH, the absorption decreased significantly with the extension of the irradiation time (Supplementary Fig. 2B). For an immediate comparison, the ABDA absorbance values as function of the irradiation time were plotted (Supplementary Fig. 2C) and for both solutions (control and sample) a good linear relationship was observed.

The heat generation of the nanoplatforms under continuous illumination was investigated (Supplementary Information). As clearly demonstrated by the thermal images acquired after an irradiation time of 90 min (Supplementary Fig. 3), in the case of the control solution (see Supplementary Information for its description), a non-significant temperature variation was observed, whereas in the case of Ir_en_-AuSiO_2__COOH, a photothermal heating of the irradiated solution as well as the surrounding environment was highlighted. The observed thermoplasmonic effect can be explained as a result of a photothermal conversion of the energy absorbed by Ir_en_ molecules and partially transferred to the metal nanoparticle. In particular, the evidenced spectral overlap between the emission band of the Iridium compound and the LSPR of the gold core, allows donor–acceptor energy transfer processes, from the Iridium-based molecules to the gold core, which in turn converts the received energy into heat [51]. Therefore, using a single excitation wavelength in the Ir_en_ absorption region, Ir_en_-AuSiO_2__COOH nanoplatforms can act as luminescent probes, photosensitizing agents, and heat nanosources.

**Fig. 2:**
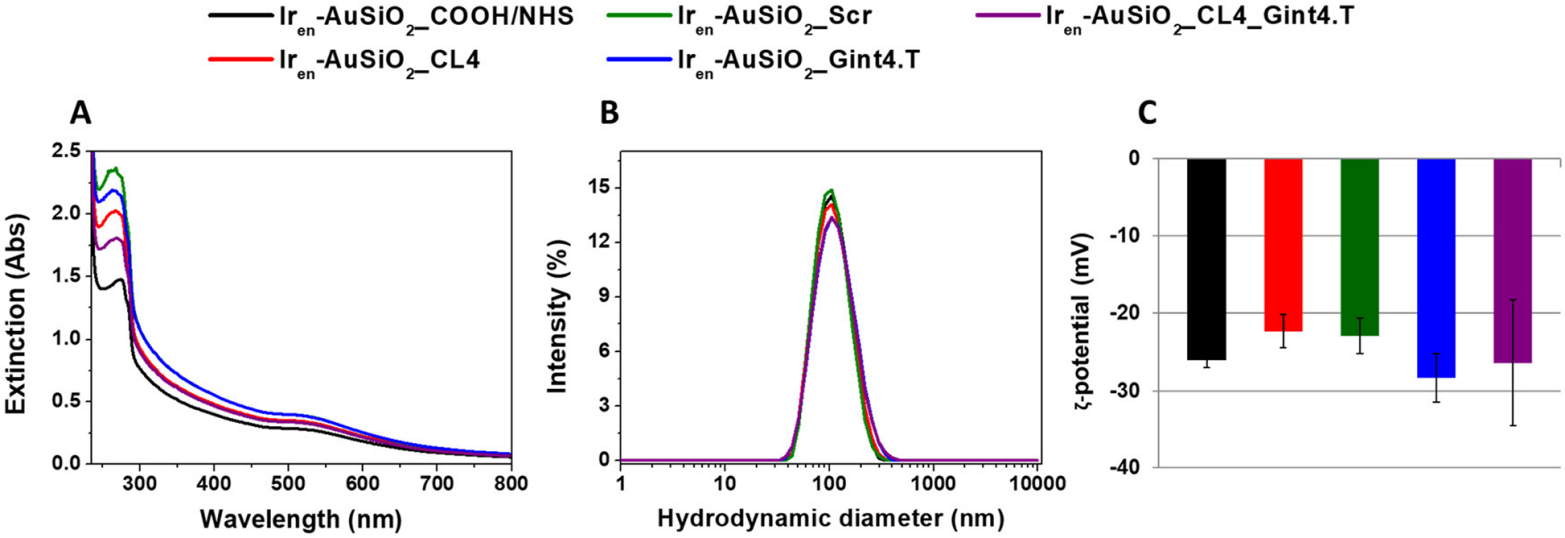
Characterization of Ir_en_-AuSiO_2__Aptamer nanoplatforms. **A** UV–Vis spectra, **B** hydrodynamic diameters, **C** ζ-potential values of Ir_en_-AuSiO_2__Aptamer nanoplatforms dispersed in water

In order to specifically address Ir_en_-AuSiO_2__COOH to target tumor/stromal cells, conjugation with the EGFR CL4 (Ir_en_-AuSiO_2__CL4) or PDGFRβ Gint4.T (Ir_en_-AuSiO_2__Gint4.T) aptamers, and dual functionalization with both CL4 and Gint4.T (Ir_en_-AuSiO_2__CL4_Gint4.T) were performed. Ir_en_-AuSiO_2__Scr, decorated with a non-targeting scrambled aptamer, were used as a negative control. The schematic representation of the synthetic steps involved in the development of the aptamers-conjugated gold–silica nanoplatforms and a key illustration of all the prepared samples is shown in Fig. 1B,C. Specifically, to obtain aptamers-nanoplatforms conjugates (Ir_en_-AuSiO_2__Aptamer), the free –COOH groups of Ir_en_-AuSiO_2__COOH were activated—through EDC/NHS chemistry [52, 53]—to react with the 5' NH_2_-aptamers, with consequent formation of amide bonds (− CO–NH −). In particular, EDC reacts with a carboxylic group on nanoparticles surface, resulting in an amine-reactive O-acylisourea intermediate. The addition of NHS stabilizes the amine-reactive intermediate by converting it to an amine-reactive NHS ester. Then, through a nucleophilic attack the NHS ester is easily displaced by the amine group on the 5′-end of aptamer to yield a stable amide bond (Fig. 1B). Extinction spectra of Ir_en_-AuSiO_2__COOH/NHS and all conjugated samples are displayed in Fig. 2A. Where the spectral profile of Ir_en_-AuSiO_2__COOH/NHS results superimposable on the extinction spectrum of Ir_en_-AuSiO_2__COOH (Supplementary Fig. 1C), in the case of Ir_en_-AuSiO_2__CL4, Ir_en_-AuSiO_2__Scr, Ir_en_-AuSiO_2__Gint4.T and Ir_en_-AuSiO_2__CL4_Gint4.T, a shoulder at 260 nm—characteristic of the maximum absorption peak of RNA sequences [54]—was observed. This absorption feature proves the successful RNA functionalization. The stability of aptamers-conjugated nanoparticles was monitored by recording extinction spectra over 7, 14, 21 and 28 days. As shown in the Supplementary Fig. 4A-D, no significant variation of the spectral profiles – decrease in optical density or shift of absorption maxima—was observed over time, highlighting the stability of the nanoplatforms in the aqueous environment.

The DLS results (Fig. 2B,C, Supplementary Table 1) reported that the Ir_en_-AuSiO_2__COOH/NHS nanoplatforms were characterized by a hydrodynamic diameter equal to 102.0 ± 0.15 nm (PDI of 0.172) and a negative ζ-potential value of -26.0 mV. Ir_en_-AuSiO_2__CL4 and Ir_en_-AuSiO_2__Scr were characterized by a hydrodynamic diameter of 102.1 ± 0.93 nm (PDI = 0.163) and 101.4 ± 0.20 nm (PDI = 0.162), respectively, and a negative ζ-potential value of -22.3 and -22.9 mV each. Ir_en_-AuSiO_2__Gint4.T and Ir_en_-AuSiO_2__CL4_Gint4.T were characterized by a hydrodynamic diameter equal to 104.4 ± 0.47 nm (PDI = 0.168) and 103.7 ± 0.82 nm (PDI = 0.162), respectively, and a negative ζ-potential value of -28.3 and -26.4 mV each. Therefore, Ir_en_-AuSiO_2__COOH/NHS as well as all Ir_en_-AuSiO_2__Aptamer samples have a hydrodynamic diameter very similar to each other. However, these values are larger than that of Ir_en_-AuSiO_2__COOH, plausibly due to variations in the surface coating and/or hydration sphere. Conversely, no significant changes were observed in the surface charge values of all the nanoplatforms (Ir_en_-AuSiO_2__COOH, Ir_en_-AuSiO_2__COOH/NHS, Ir_en_-AuSiO_2__Aptamer), as result in all cases of an extensive presence of free carboxyl groups on the surface of the nanoplatforms. The amount of each aptamer conjugated to the nanoplatforms was evaluated by RT-qPCR analysis on Ir_en_-AuSiO_2__Aptamer (Supplementary Fig. 5). We quantified approximately 3.0 pmol aptamer per 16.0 pmol of Ir_en_-AuSiO_2__ Aptamer nanoplatform and the efficiency of conjugation ranged between 2 and 6% with a mean ± SEM equal to 3.3 ± 0.7%.

### 2D cell imaging by Ir_en_-AuSiO_2__Aptamer nanoplatforms

To assess the cell targeting/uptake ability of nanoparticles conjugated to a single aptamer, CL4 or Gint4.T, or functionalized to both aptamers, we took advantage of different human cell lines expressing only EGFR, only PDGFRβ, both, or neither of the receptors. Specifically, human MES-TNBC MDA-MB-231 and BT-549 cells were chosen as double-positive cells as they express both EGFR and PDGFRβ ([23] and Supplementary Fig. 6) and, consequently, are specifically targeted by the two aptamers either when grown in classical 2D cultures and 3D Matrigel-embedded cultures, or implanted in mice [23, 30, 39]. Furthermore, we previously proved that CL4 strongly improves the uptake of drug-loaded and aptamer-decorated poly(lactic-co-glycolic)-poly ethylene glycol-based nanoparticles into both cell lines in vitro and in vivo [24]. As models of EGFR^+^/PDGFRβ^−^ cell lines, we used breast cancer BT-474 and epidermoid carcinoma A431 cell lines, which express moderate and high levels of EGFR, respectively, without expressing PDGFRβ ([23, 44] and Supplementary Fig. 6), and are thus recognized by CL4 [19] but not Gint4.T [35, 39]. Moreover, stromal MSCs that express high levels of PDGFRβ were selected as EGFR^−^/PDGFRβ^+^ cells ([30, 55] and Supplementary Fig. 6). Importantly, we previously demonstrated that Gint4.T, by blocking PDGFRβ, efficiently inhibits MSCs recruitment into TNBC thus preventing their pro-metastatic function [30]. Finally, EGFR^−^/PDGFRβ^−^ breast cancer MCF7 cells ([23] and Supplementary Fig. 6) were used as a negative control.

**Fig. 3:**
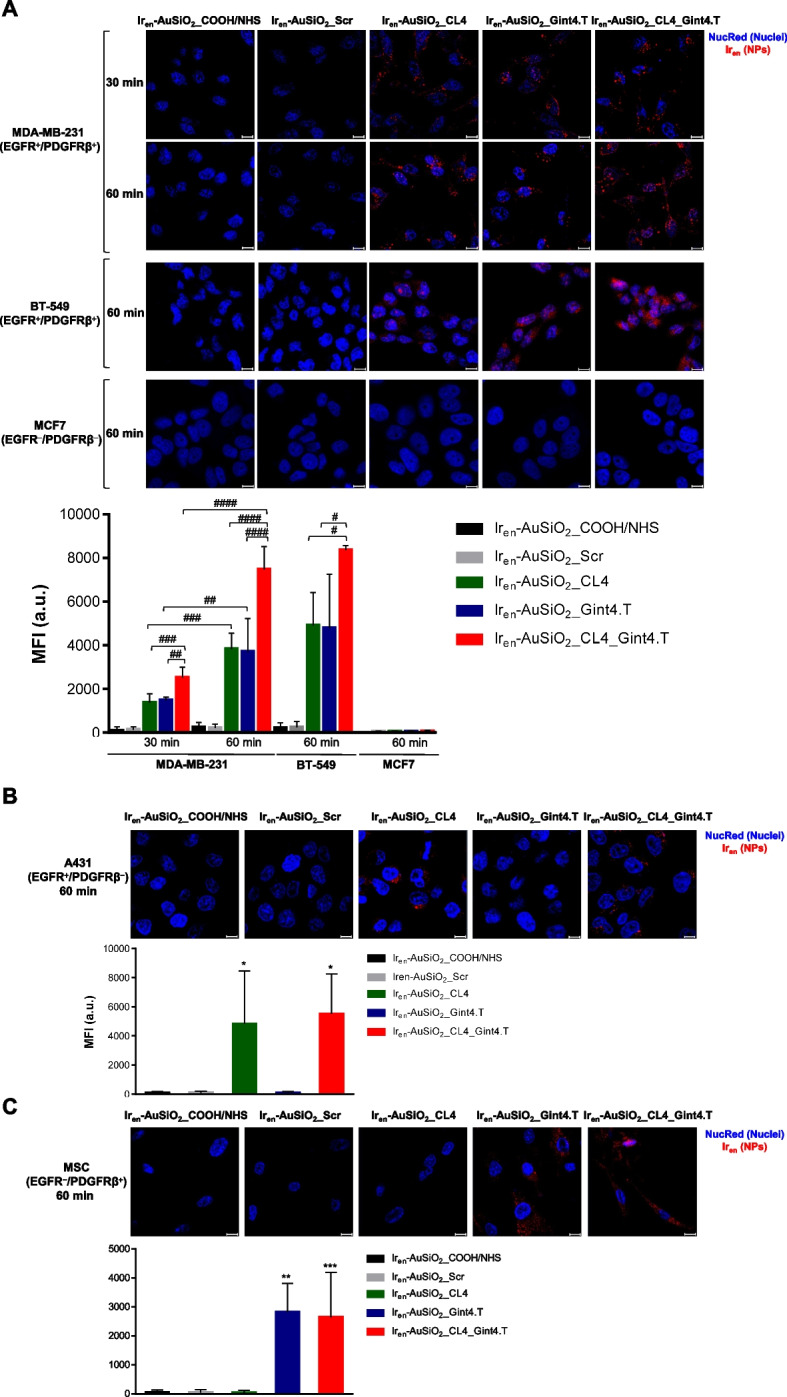
Selective cell uptake of CL4 and/or Gint4.T-decorated Ir_en_-AuSiO_2__Aptamer nanoplatforms in 2D cultures. Representative confocal images of **A** MDA-MB-231 and BT-549 (EGFR^+^/PDGFRβ^+^), and MCF7 (EGFR^−^/PDGFRβ^−^) cells, **B** A431 (EGFR^+^/PDGFRβ^−^) cells, and **C** MSCs (EGFR^−^/PDGFRβ^+^) cells, incubated with Ir_en_-AuSiO_2__Aptamer or unconjugated Ir_en_-AuSiO_2__COOH/NHS nanoparticles at 37 °C for the indicated times. After washing and fixation, cells were labeled with NucRed (blue) to stain nuclei, and nanoparticles are displayed in red. Magnification: 63 × , 1.0 × digital zoom, scale bar = 10 μm. All digital images were captured under the same settings to enable a direct comparison of staining patterns. **A**-**C** Mean fluorescence intensity (MFI) was quantified using Zeiss software on at least ten separate images for each condition. Bars depict means ± SD (*n* = 3). *p < 0.05, **p < 0.01, ***p < 0.001 relative to Ir_en_-AuSiO_2__Scr; #*p* < 0.05, ##*p* < 0.01, ###*p* < 0.001, ####*p* < 0.0001

The intrinsic Iridium-compound associated fluorescence emitted (Em = 520 nm) from the unconjugated Ir_en_-AuSiO_2__COOH/NHS or Ir_en_-AuSiO_2__Aptamer nanoplatforms (5 μM Ir_en_ concentration) was collected by confocal microscopy after incubation of the cells with the nanoparticles at 37 °C in the presence of yeast tRNA and salmon sperm DNA competitors to hinder any non-specific interactions. As shown (Fig. 3A), the signal associated with Ir_en_-AuSiO_2__Aptamer (Ir_en_-AuSiO_2__CL4, Ir_en_-AuSiO_2__Gint4.T and Ir_en_-AuSiO_2__CL4_Gint4.T) nanoparticles was clearly visible in the cytoplasm of MDA-MB-231 cells at 30 min and further increased at 60 min of incubation. Conversely, an almost undetectable signal was obtained with unconjugated Ir_en_-AuSiO_2__COOH/NHS or scrambled decorated Ir_en_-AuSiO_2__Scr nanoparticles at the two incubation times. Importantly, MDA-MB-231 cells treated with the dual-targeting EGFR/PDGFRβ nanoparticles had significantly higher fluorescence intensity than that treated with single-targeting EGFR or PDGFRβ nanoparticles (Fig. 3A), indicating that CL4 and Gint4.T, simultaneously attached to the nanoparticles, confer improved cellular uptake. Similar results were observed on EGFR^+^/PDGFRβ^+^ BT-549 cells (Fig. 3A). Labeling cells with WGA to visualize cell membrane confirmed that the CL4 and Gint4.T aptamers properly drive the nanoparticles in the cytoplasm (Supplementary Fig. 7A). As expected, no signal was observed in double negative MCF7 control cells (Fig. 3A).

As a next step, the nanoparticle’ formulations were incubated for 60 min onto only EGFR^+^ A431 (Fig. 3B) and BT-474 (Supplementary Fig. 7B) cell lines, and only PDGFRβ^+^ MSCs (Fig. 3C), and confocal microscopy analyses revealed that the internalization ability of the nanoparticles strictly depends on the aptamer present on the nanoparticle’s surface and the expression of its receptor partner on the target cell.

Overall, these results show efficient capability of Ir_en_-AuSiO_2__Aptamer nanoparticles to selectively enter into the cell through EGFR and/or PDGFRβ recognition and indicate that the carriers’ parameters, including composition, size, shape and surface chemistry, do not affect the interactions of CL4 and Gint4.T aptamers to their proper receptor on membranes of target cells.

### Uptake of Ir_en_-AuSiO_2__Aptamer nanoplatforms in 3D multicellular tumor spheroids

Next, we wondered whether multifunctional nanoparticles retain their targeting ability in more relevant in vitro cancer models by using 3D multicellular spheroids obtained by co-culturing tumor and stromal cells on non-adhesive culture dishes, which resemble the organization and properties of a native tumor, as a key factor of translational medicine [56, 57]. Even if these models have been successfully used to study tumor-MSC interaction [56, 57], to date a still limited number of studies have employed 3D tumor spheroids for evaluating the functionality of nanomedicine [58].

**Fig. 4:**
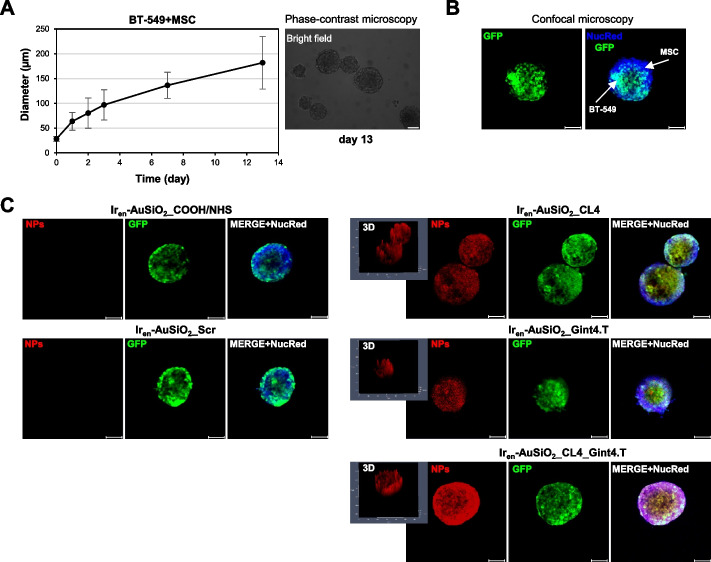
Selective uptake of CL4 and/or Gint4.T-decorated Ir_en_-AuSiO_2__Aptamer nanoplatforms in 3D heterotypic spheroids. **A** (left) Growth kinetic of BT-549-GFP + MSC spheroids represented in spheroid diameter over 13 days. The representative phase-contrast microscopy images of spheroids formation over the course of seven days are reported in Supplementary Fig. 8A. Data are presented as the mean ± SD (*n* = 3); (right) representative phase-contrast microscopy image of the spheroids grown at day 13. Magnification: 10 × , scale bar = 100 μm. **B** Representative confocal image of the heterotypic spheroid at day 13. BT-549-GFP cells are visualized in green and nuclei, stained with NucRed 647, in blue. White arrows in the merged images highlight the mixed composition of the spheroid (MSC, blue; BT-549-GFP, blue light). **C** Representative confocal images of BT-549-GFP/MSC spheroids grown at day 13 and then incubated with Ir_en_-AuSiO_2__CL4, Ir_en_-AuSiO_2__Gint4.T, Ir_en_-AuSiO_2__CL4_Gint4.T, Ir_en_-AuSiO_2__Scr or unconjugated Ir_en_-AuSiO_2__ COOH/NHS for 24 h at 37 °C. Nanoparticles, BT-549-GFP cells and nuclei are displayed in red, green and blue, respectively. 3D images are shown. **B**,**C** Magnification: 10 × , 1.0 × digital zoom, scale bar = 100 μm. All digital images were captured under the same settings to enable a direct comparison of staining patterns

In order to distinguish cancer cells from stromal cells we established tumor spheroids consisting of GFP-labeled BT-549 and unlabeled MSCs. In agreement with previous findings, both BT-549 cells [59, 60] and MSCs [61, 62] were able to form spheroids alone in non-attached culture (Supplementary Fig. 8A). When cancer cells were co-cultured with stromal cells at a 1:4 ratio, respectively [63], consistent heterotypic spheroids (BT-549 + MSCs), were obtained, reaching approximately 180 µm in diameter, in 13 days (Supplementary Fig. 8A and Fig. 4A). In order to better visualize formed spheroids, they were embedded in Matrigel and observed by confocal microscopy (Fig. 4B). NucRed 647 nuclear stain was used to visualize both cancer and stromal cells, while the GFP to visualize cancer cells. As shown (Fig. 4B), the presence of NucRed 647 nuclear stain (visualized in blue) either associated to the GFP signal (BT-549) or not (MSCs) (see arrows in the merged image), indicates the mixed composition of the spheroids. Moreover, immunofluorescence with α-SMA and FAP markers confirmed the presence of tumor-associated MSCs in the heterotypic spheroids (Supplementary Fig. 8B). To examine the penetration of the different nanoformulations into the spheroids, BT-549/MSCs spheroids were exposed to Ir_en_-AuSiO_2__Aptamer and unconjugated nanoparticles, at an Ir_en_ concentration of 5 μM, for 24 h at 37 °C and visualized by confocal microscopy (Fig. 4C). Importantly, the presence of the EGFR and PDGFRβ targeting aptamers on the surface of the nanoparticles, either single-targeted (Ir_en_-AuSiO_2__CL4 and Ir_en_-AuSiO_2__Gint4.T) or dual-targeted (Ir_en_-AuSiO_2__CL4_Gint4.T), allowed them to penetrate the mixed tumor/stromal spheroids as qualitatively displayed by the nanoplatform-associated fluorescent signal (visualized in red) that was visible throughout the spheroids. 3D images clearly indicated the accumulation of the aptamer-functionalized nanoparticles inside the spheroid mass. Conversely, no signal was detected with unconjugated Ir_en_-AuSiO_2_ or Ir_en_-AuSiO_2__Scr negative control (Fig. 4C), thus indicating that passive infiltration of the spheroids by untargeted nanoparticles could not occur at least under the experimental conditions used.

### Anticancer photokilling activity of Ir_en_-AuSiO_2__Aptamer nanoplatforms in 3D multicellular tumor spheroids

We wondered whether the nanoparticles conjugated to CL4, Gint4.T or both the aptamers, once penetrated into the spheroids, kill cancer and stromal cells upon light irradiation. Thus, we first validated that AuSiO_2__COOH/NHS, AuSiO_2__Scr, AuSiO_2__CL4 or AuSiO_2__Gint4.T nanoparticles, which had no load of photosensitizing and luminescent molecule Ir_en_, when incubated for 24 h at 37 °C on MDA-MB-231 and BT-549 cells have no adverse effects on cell viability (Supplementary Fig. 9A,B).

Next, before moving on to more complex 3D cell systems, we verified the therapeutic efficacy of Ir_en_-loaded nanoformulations in 2D cell cultures. To this aim, BT-549 and MDA-MB-231, positive for EGFR and PDGFRβ, BT-474 and A431, positive for EGFR, MSCs, positive for PDGFRβ, and MCF7 cells, negative for both receptors, were incubated with the different nanoformulations containing Ir_en_ (5 µM) and decorated or not with the aptamers, for 1 h, given the rapid cell uptake of aptamer-decorated nanoparticles, washed to remove not-internalized nanoparticles, exposed to 1-h light irradiation and analyzed for their viability after 24 h. As shown (Supplementary Fig. 9C-H), Ir_en_-AuSiO_2__Aptamer nanoplatforms demonstrated efficient capability to selectively kill the cells, through EGFR and/or PDGFRβ recognition, in comparison with untargeted NPs (unconjugated Ir_en_-AuSiO_2__ COOH/NHS or scrambled-conjugated Ir_en_-AuSiO_2__Scr). No toxicity of aptamer-decorated nanoplatforms was observed on each cell line under dark conditions, thus indicating their safety behavior at least in the concentrations tested in the PDT experiments. We verified that free Ir_en_ is nontoxic and does not contribute to the photoinduced killing activity effects, at least at the concentration and exposure time used in the encapsulated form (Supplementary Fig. 9C-H).

**Fig. 5:**
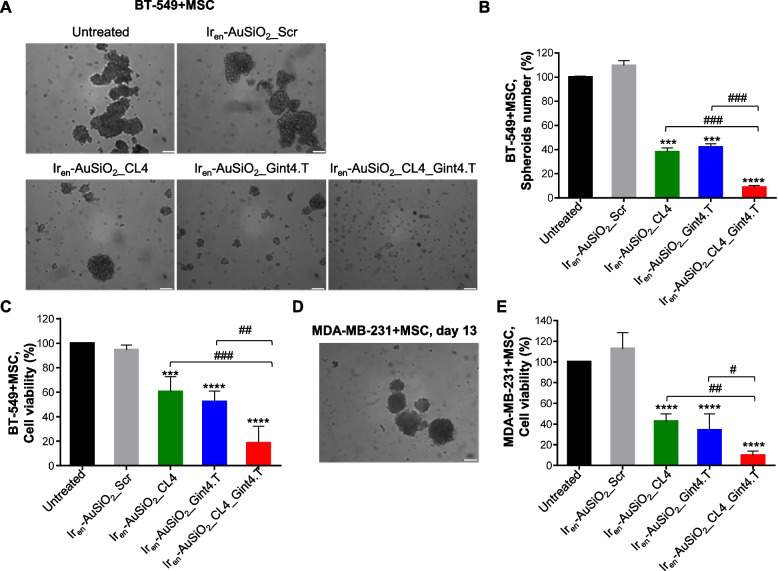
Anticancer activity of Ir_en_-AuSiO_2__Aptamer nanoplatforms on 3D spheroids of EGFR^+^/PDGFRβ^+^ cancer cells and MSC. **A** (left) Representative phase-contrast microscopy images of BT-549/MSC spheroids treated with Ir_en_-AuSiO_2__CL4, Ir_en_-AuSiO_2__Gint4.T, Ir_en_-AuSiO_2__CL4_Gint4.T or untargeted Ir_en_-AuSiO_2__Scr. Spheroids treatment with specific aptamer-decorated nanoplatforms, but not with Ir_en_-AuSiO_2__Scr, inhibits both the** B** number of spheroids and **C** cell viability, expressed as percentage of viable treated cells with respect to untreated spheroids. **D** Representative phase-contrast microscopy image of MDA-MB-231/MSC spheroids grown at day 13. **E** Cell viability assay on MDA-MB-231/MSC spheroids treated as in A. **A**,**D** Magnification: 10 × , scale bar = 100 μm. **B**,**C**,**E** Bars depict mean ± SD (*n* = 3). ***p < 0.001, ****p < 0.0001 relative to Ir_en_-AuSiO_2__Scr; #*p* < 0.05, ##*p* < 0.01, ###*p* < 0.001. No statistically significant variations among Ir_en_-AuSiO_2__Scr and untreated were obtained

Importantly, when incubated for 24 h with heterotypic BT-549 + MSCs spheroids, Ir_en_-AuSiO_2__CL4, Ir_en_-AuSiO_2__Gint4.T and Ir_en_-AuSiO_2__CL4_Gint4.T (5 µM Ir_en_ concentration; 1-h light irradiation), disrupted spheroid structure (Fig. 5A,B) and inhibited cell viability (Fig. 5C), as determined by spheroids number counting and CellTiter-Glo 3D cell viability assay, respectively, with the dual targeted nanoparticles being more effective than the single-targeted ones (approximately 80% inhibition, Ir_en_-AuSiO_2__CL4_Gint4.T *vs* 40% inhibition, Ir_en_-AuSiO_2__CL4, and 50% inhibition, Ir_en_-AuSiO_2__Gint4.T). Conversely, no effect was observed on controls consisting of untreated or treated with scrambled-decorated Ir_en_-AuSiO_2_ nanoformulations spheroids (Fig. 5A-C). Similarly, a higher effect on cell viability inhibition was observed upon treatment of heterotypic MDA-MB-231 + MSCs spheroids with Ir_en_-AuSiO_2__CL4_Gint4.T compared to single-targeted nanoparticles (Fig. 5D,E).

**Fig. 6:**
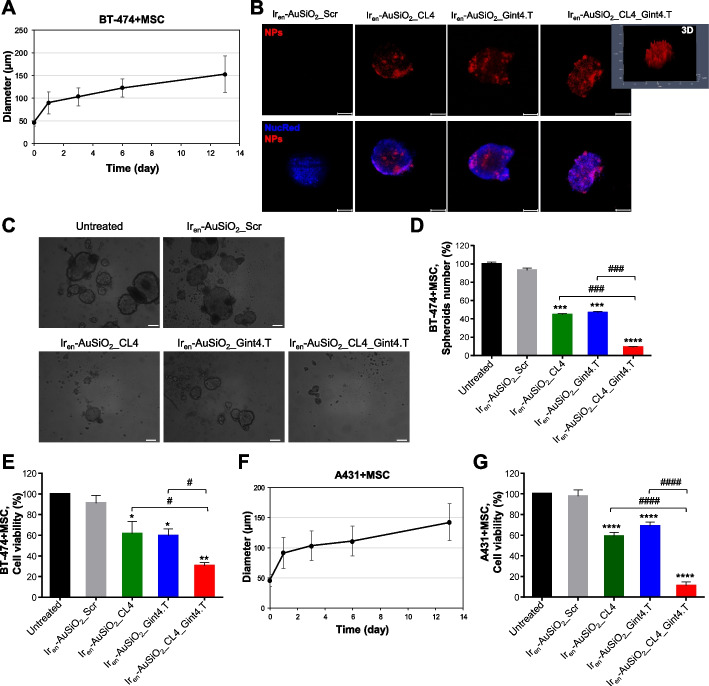
Anticancer activity of Ir_en_-AuSiO_2__Aptamer nanoplatforms on 3D spheroids of EGFR^+^/PDGFRβ^−^ cancer cells and MSC. **A** Growth kinetic of BT-474 + MSC spheroids represented in spheroid diameter over 13 days. The representative phase-contrast microscopy images of spheroids formation over the course of thirteen days are reported in Supplementary Fig. 10A. **B** Representative confocal images of BT-474/MSC spheroids grown at day 13 and then incubated with Ir_en_-AuSiO_2__CL4, Ir_en_-AuSiO_2__Gint4.T, Ir_en_-AuSiO_2__CL4_Gint4.T, or untargeted Ir_en_-AuSiO_2__Scr for 24 h at 37°C. Nanoparticles and nuclei are displayed in red and blue, respectively. 3D image (Ir_en_-AuSiO_2__CL4_Gint4.T) is shown. Magnification: 10 × , 1.0 × digital zoom, scale bar = 100 μm. All digital images were captured under the same settings to enable a direct comparison of staining patterns. **C** Representative phase-contrast microscopy images of BT-474/MSC spheroids treated as indicated. Spheroids treatment with specific aptamer-decorated nanoplatforms, but not with Ir_en_-AuSiO_2__Scr, inhibits both the **D** number of spheroids and **E** cell viability, expressed as percentage of viable treated cells with respect to untreated spheroids. **F** Growth kinetic of A431 + MSC spheroids represented in spheroid diameter over 13 days. The representative phase-contrast microscopy images of spheroids formation over the course thirteen days are reported in Supplementary Fig. 10B. **G** Cell viability assay on A431 + MSC spheroids treated as in C. **A**,**D**,**E**,**F**,**G** Data are presented as the mean ± SD (*n* = 3); *p < 0.05, **p < 0.01, ***p < 0.001, ****p < 0.001 relative to Ir_en_-AuSiO_2__Scr; #*p* < 0.05, ###*p* < 0.001, ####*p* < 0.0001. No statistically significant variations among Ir_en_-AuSiO_2__Scr and untreated were obtained

Because MES-TNBC cells express both EGFR and PDGFRβ, in order to confirm the efficacy of the dual-targeting nanovectors on both cancer and stromal cells, we generated tumor spheroids consisting of cancer BT-474 cells (only EGFR^+^) and MSCs (only PDGFRβ^+^) that grew as colonies with approximately a 150 μm diameter after 13 days in culture (Supplementary Fig. 10A and Fig. 6A). As shown, uptake into BT-474/MSCs spheroids of either single-targeted Ir_en_-AuSiO_2__CL4 and Ir_en_-AuSiO_2__Gint4.T, as well as dual-targeted Ir_en_-AuSiO_2__CL4_Gint4.T nanoparticles after 24 h of incubation was clearly detected by confocal microscopy (Fig. 6B) and the cytotoxic effect of Ir_en_-AuSiO_2__CL4_Gint4.T nanoparticles on both the spheroid disruption (Fig. 6C,D) and cell viability inhibition (Fig. 6E) was higher than that of either Ir_en_-AuSiO_2__CL4 or Ir_en_-AuSiO_2__Gint4.T (approximately 70% inhibition for Ir_en_-AuSiO_2__CL4_Gint4.T *vs* 40% inhibition for Ir_en_-AuSiO_2__CL4 and Ir_en_-AuSiO_2__Gint4.T, relative to untreated cultures), thus confirming the ability of dual-decorated nanoparticles to kill both cancer and stromal cells through EGFR and PDGFRβ targeting, respectively.

Similarly, tumor spheroids consisting of A431 cells (only EGFR^+^) grown with MSCs (only PDGFRβ^+^) up to 13 days (Supplementary Fig. 10B and Fig. 6F) were higher affected by EGFR/PDGFRβ bispecific nanoformulations than those single-targeted, with a reduction of cell viability of approximately 90% for Ir_en_-AuSiO2_CL4_Gint4.T, 40% for Ir_en_-AuSiO_2__CL4 and 30% for Ir_en_-AuSiO_2__Gint4.T relative to untreated cultures (Fig. 6G).

### Anticancer photokilling activity of Ir_en_-AuSiO_2__Aptamer nanoplatforms on 3D patient-derived cancer organoids

**Fig. 7:**
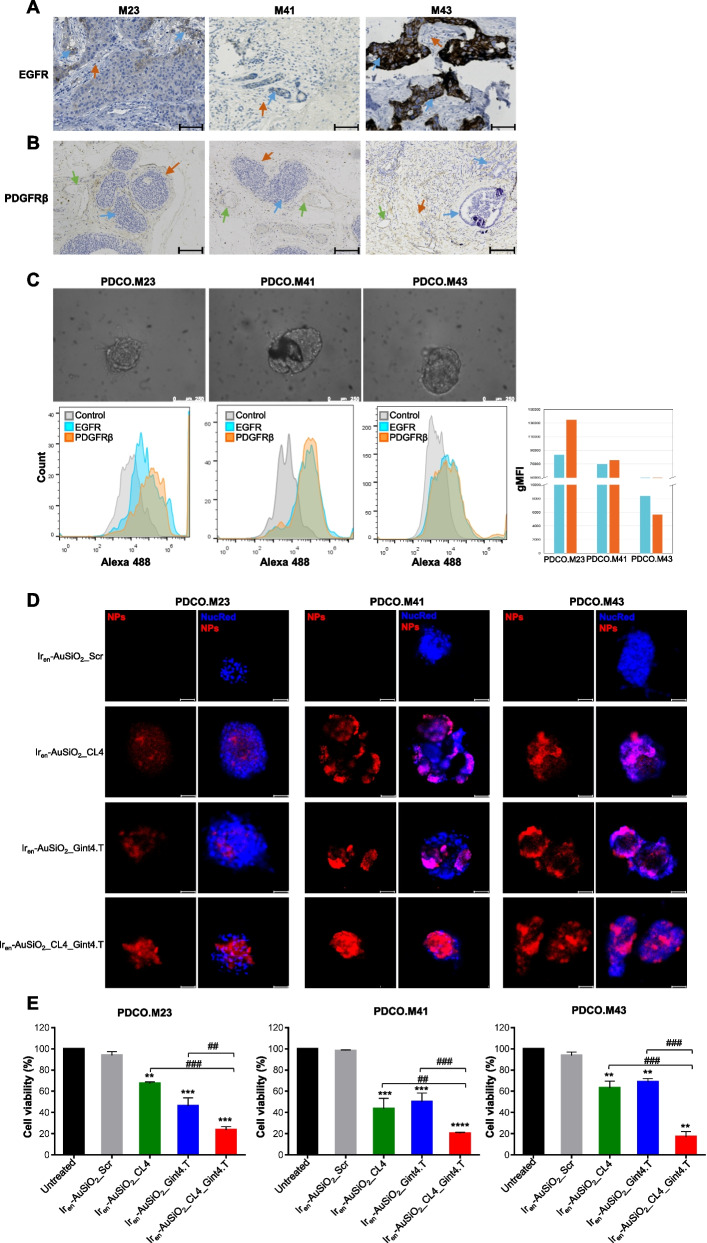
Anticancer activity of Ir_en_-AuSiO_2__Aptamer nanoplatforms on 3D patient-derived breast cancer organoids. Representative images of three breast cancer samples (M23, M41 and M43) stained for **A** EGFR and **B** PDGFRβ. In **A**, the blue arrows indicate EGFR-positive neoplastic cells (M23, moderate membrane expression; M41, mild membrane expression; M43, strong membrane expression); the red arrows indicate EGFR-negative peritumoral stromal cells. Magnification: 10 × , scale bar = 100 μm. In **B**, the blue arrows indicate PDGFRβ-negative neoplastic cells; the orange arrows indicate PDGFRβ-positive peritumoral stromal cells (M23 and M41, mild cytoplasmic expression; M43, moderate cytoplasmic expression); the green arrows indicate the endothelial cells of vessels (red blood cells are visible inside) positive for PDGFRβ. Magnification: 5 × , scale bar = 50 μm. **C** (upper) Representative phase-contrast microscopy images of PDCOs obtained by M23, M41 and M43 tumor samples, magnification: 20 × , scale bar = 250 μm; (lower) flow cytometry analyses to confirm the expression of EGFR and PDGFRβ in the three PDCOs. The histogram indicates the geometric mean fluorescence intensity (gMFI) of EGFR and PDGFRβ expressed on PDCOs, calculated using FlowJo software. **D** Representative confocal images of PDCO.M23, PDCO.M41 and PDCO.M43 incubated with Ir_en_-AuSiO_2__CL4, Ir_en_-AuSiO_2__Gint4.T, Ir_en_-AuSiO_2__CL4_Gint4.T or untargeted Ir_en_-AuSiO_2__Scr for 24 h at 37 °C. Nanoparticles and nuclei are displayed in red and blue, respectively. Magnification: 10 × , 2.0 × digital zoom, scale bar = 50 μm. All digital images were captured under the same settings to enable a direct comparison of staining patterns. **E** Cell viability assay on PDCO.M23, PDCO.M41 and PDCO.M43 treated as indicated. Bars depict mean ± SD of two independent experiments performed in triplicate. **p < 0.01, ***p < 0.001, ****p < 0.001 relative to Ir_en_-AuSiO_2__Scr; ##*p* < 0.01, ###*p* < 0.001, ####*p* < 0.0001. No statistically significant variations among Ir_en_-AuSiO_2__Scr and untreated were obtained

To further prove the targeting efficacy and the photoinduced killing activity of dual aptamer-decorated nanoparticles, through selective recognition of EGFR-positive tumor cells and PDGFRβ-positive stromal component in the entire tumor bulk, we employed 3D patient organoids from human surgical specimens collected from three patients with diagnosis of breast cancer. Tumor samples, henceforth named M23, M41 and M43, were chosen for the presence of EGFR only in tumor cells (Fig. 7A, blue arrows) and PDGFRβ only in the stromal component (Fig. 7B), specifically in vascular endothelial cells (green arrows) and/or mesenchymal stromal cells (orange arrows), as assessed by immunohistochemical analyses.

The clinical pathological characteristics of the three tumors are summarized in the Supplementary Table 2. PDCOs at the first passage were cultured up to day 10 (Fig. 7C, upper panels), disaggregated into a single-cell suspension and analyzed by flow cytometry to confirm the expression of EGFR on epithelial tumor cells and PDGFRβ on stromal cells (Fig. 7C, lower panels). Confirming results obtained in 3D multicellular tumor spheroids, Ir_en_-AuSiO_2__Aptamer nanoplatforms efficiently spread into the organoid mass (Fig. 7D) and exert excellent anticancer photokilling activity, which was superior with the dual-aptamer-decorated nanoparticles over single-aptamer-conjugated (Fig. 7E). Conversely, no effect was observed in untreated organoids or those treated with scrambled-aptamer-conjugated nanoparticles (Fig. 7E).

Taken together, our results show the striking antitumor potential of our bispecific light-triggered nanoplatforms targeting tumor and stromal cells and highlight the potential translational value of integrative research combining patient-derived organoids and cancer nanomedicine.

## Discussion

In breast cancer development, as in many other human tumors, stromal cells contribute to the establishment of a supportive microenvironment for tumor cells. The TME, rich in stromal components, can contribute to treatment resistance and limit the effectiveness of therapies targeting tumor cells alone [1]. Therefore, therapies that take into account the dynamic interplay between stroma and tumor cells are gaining attention.

Here we focus on the design and evaluation of bispecific nanotherapeutics that selectively act on both tumor and stromal cells, presenting a promising strategy to advance the outcome of traditional tumor cell-targeting nanomedicine by influencing the tumor-supporting activity of stromal cells in the complex TME. To this aim we have employed oligonucleotide aptamers as cell targeting agents because of their stability, ease of manufacture at high reproducibility, and low or absent immunogenicity. Their chemical synthesis and versatility for chemical modification allow easier conjugation to different kinds of nanoplatforms, at higher yield and lower costs, than other form of ligands as antibodies or peptides [28, 64], which is relevant for industrial scale up. To date, a limited number of dual-aptamer modified nanoplatforms have been developed for achieving cancer therapeutic efficacy superior to that of single targeting through the recognition of different tumor cell types [65, 66] or different receptors on the same tumor cells [67, 68] and, to the best of our knowledge, this is the first study exploring bispecific nanoparticles with two different aptamers for exerting excellent anti-tumor cytotoxic effects on both tumor cells and the reactive stroma.

We prepared gold-core/silica-shell nanoparticles via reverse microemulsion method, an ideal synthetic approach to get spherical and highly monodispersed particles. The presence of the Iridium compound in the polysiloxane matrix gives the nanostructure photosensitizing and luminescent capabilities, while that of the gold-core ensures photothermal properties. The nanoparticle surface containing carboxyl groups was decorated with amino-terminated EGFR (CL4) and PDGFRβ (Gint4.T) aptamers, through the formation of amide bonds, creating a multifunctional nanoplatform termed Ir_en_-AuSiO_2__CL4_Gint4.T. The specificity and synergistic effects of these dual-aptamer-decorated nanoparticles were rigorously assessed through confocal microscopy and cell viability assays on various human cell types, including TNBC cell lines, luminal/HER2-positive breast cancer cells, epidermoid carcinoma cells, and adipose-derived mesenchymal stromal/stem cells, and preclinical 3D stroma-rich breast cancer models, consisting of either 3D spheroids cocultures of tumor cells and MSCs, and breast cancer organoids derived from pathologically and molecularly well-characterized human tumors. Crucially, the results demonstrate the efficient capability of aptamer-conjugated nanoplatforms not only to selectively enter target cells and induce cell death in 2D cell cultures but also uniformly spread into both breast cancer spheroids and organoids and disrupt them through recognition of EGFR^+^ tumor cells and PDGFRβ^+^ stromal cells, highlighting the superiority of dual-aptamer-decorated nanoparticles over single-aptamer-conjugated counterparts.

Notably, EGFR is a well characterized surface molecule for epithelial cells in many types of cancers and, accordingly, we and other groups worldwide have successfully applied the EGFR CL4 aptamer as tumor ligand to decorate nanocarriers actively targeted to TNBC [24–27, 69, 70], hepatocellular carcinoma [68], osteosarcoma [65] and chordoma [71]. Based on these observations, our bispecific Ir_en_-AuSiO_2__CL4_Gint4.T nanoparticles could be safely applied not only to breast cancer PDO, as in the present study, but also several others EGFR-expressing cancers. At the same time, the proposed strategy for aptamer-functionalized nanoformulation can be easily adapted to different targets by switching the aptamers for other malignant tumors.

Also, we proved that the Gint4.T aptamer, by binding to PDGFRβ expressed both on tumor cells and immune populations enhances the efficacy of anti-programmed cell death-ligand 1 monoclonal antibodies in inhibiting tumor growth and metastasis formation in a syngeneic TNBC mouse model [35]. Thus, immuno- and targeted therapy may be easily combined with our aptamer-targeted nanomedicine for synergistically targeting either tumor and immune cells.

Moreover, in the context of highly aggressive human cancers, including MES-TNBC [39, 72] and different glioblastoma (GBM) subtypes [73], being characterized by a strong PDGFRβ-positivity on tumor cells and self-renewing stem cells, Ir_en_-AuSiO_2__CL4_Gint4.T may act in a synergistic way by targeting either EGFR and PDGFRβ on the epithelial tumor cells and PDGFRβ in the stroma components. At this regards, different therapeutic nanoformulations have been reported for GBM targeting that exploit Gint4.T aptamer as a potent ligand with the capability of not only passing through the blood–brain barrier by transcytosis but also recognizing the cancer cell membrane [40, 74–76].

One important result of this study is the absence of cell toxicity of our aptamer-decorated nanoformulations in the absence of light irradiation in either 2D cell cultures and in 3D systems, including both heterotypic spheroids and breast cancer patient organoids, thus suggesting them as an effective and safe anti-tumor strategy in clinical settings. Further investigation in humanized mice models will be performed to assess the pharmacokinetic and pharmacodynamic profile of the proposed nanomedicine along with their therapeutic efficacy, opening new avenues for innovative therapeutics in the realm of personalized medicine.

## Conclusions

The dynamic interplay between the tumor cells and the stroma is critical for promoting tumor growth and progression, and dictate resistance to therapies. In summary, we have proposed new dual aptamer-equipped nanoformulations that combine anticancer and anti-stroma targeting for precision phototherapeutic applications. In particular, the presence of the aptamers as active targeting ligands ensures a selective addressing and the external stimuli-mediated triggering of the nanosystem provides a spatio-temporal control of the cytotoxic action for a highly selective multimodal cancer treatment approach. Albeit biodistribution and efficacy of the nanoplatforms need to be addressed in vivo, our results clearly indicate their high potential as efficient tools for precision phototherapies of breast cancers with overexpression of EGFR and likely other human tumors.

### Supplementary Information

**Additional file 1: Supplementary Figure 1.** Characterization of Iren-AuSiO2_COOH nanoparticles. **Supplementary Figure 2.** Light-induced singlet oxygen generation of Iren-AuSiO2_COOH. **Supplementary Figure 3.** Light-induced photothermal effect of Iren-AuSiO2_COOH. **Supplementary Figure 4.** Extinction spectra acquired in the time range 0-28 days of aptamers-conjugated nanoparticles dispersed in water. **Supplementary Table 1.** Properties of aptamers-nanoplatforms conjugates. **Supplementary Figure 5.** Quantification of the amount of aptamer conjugated to Iren-AuSiO2_COOH/NHS. **Supplementary Figure 6.** Expression of PDGFRβ and EGFR in different human cell lines. **Supplementary Figure 7.** Selective cell uptake of CL4 and/or Gint4.T-decorated Iren-AuSiO2_Aptamer nanoplatforms in 2D BT-549 or BT-474 cultures. **Supplementary Figure 8.** Formation of 3D spheroids of BT-549 cells and MSC. **Supplementary Figure 9.** Photodynamic effect of nanoplatforms in 2D cell cultures. **Supplementary Figure 10.** Formation of 3D spheroids of EGFR^+^/PDGFRβ^−^ cancer cells and MSC. **Supplementary Table 2.** Clinicopathological features of three selected tumor samples.

## Data Availability

All data analyzed during this study are included in this manuscript.

## Abbreviations

2D: Two-Dimensional
2′F-Py: 2′-Fluoro-pyrimidines
3D: Three-Dimensional
α-SMA: A-Smooth Muscle Actin
ABDA: 9,10-Anthracenediyl-bis(methylene)dimalonic acid
APTES: (3-Aminopropyl)triethoxysilane
BSA: Bovine Serum Albumin
DLS: Dynamic Light Scattering
DMEM: Dulbecco's Modified Eagle Medium
DPBS: Dulbecco’s Phosphate Buffered Saline
EDC: N-(3-dimethylaminopropyl)-N′-ethylcarbodiimide hydrochloride
EGFR: Epidermal Growth Factor Receptor
FAP: Fibroblast Activation Protein
GBM: Glioblastoma
GFP: Green Fluorescent Protein
HER2: Human Epidermal Growth Factor Receptor 2
Ir_en_: Iridium(III) compound ethylenediamine
LSPR: Localized Surface Plasmon Resonance
MES: Mesenchymal Subtype
Mesna: Sodium 2-mercaptoethanesulfonate
MFI: Mean fluorescence intensity
MSCs: Adipose-derived Mesenchymal Stromal/Stem cells
NHS: N-hydroxysuccinimide
PBS: Phosphate Buffer Solution
PDCOs: Patient-Derived Cancer Organoids
PDGFRβ: Platelet-Derived Growth Factor Receptor β
PDI: Polydispersity Index
PDT: Photodynamic Therapy
PTT: Photothermal Therapy
RT: Room Temperature
RT-qPCR: Reverse Transcription-Quantitative Polymerase Chain Reaction
TEM: Transmission Electron Microscopy
TEOS: Tetraethoxysilane
TME: Tumor Microenvironment
TNBC: Triple Negative Breast Cancer
WGA: Wheat Germ Agglutinin
WM: Working Medium
W/O: Water/Oil
