# Correction: Bispecifc aptamer-decorated and light-triggered nanoparticles targeting tumor and stromal cells in breast cancer derived organoids: implications for precision phototherapies

**DOI:** 10.1186/s13046-024-03159-9

**Published:** 2024-08-23

**Authors:** Simona Camorani, Alessandra Caliendo, Elena Morrone, Lisa Agnello, Matteo Martini, Monica Cantile, Margherita Cerrone, Antonella Zannetti, Massimo La Deda, Monica Fedele, Loredana Ricciardi, Laura Cerchia

**Affiliations:** 1 Institute of Experimental Endocrinology and Oncology “Gaetano Salvatore”, National Research Council, 80131 Naples, Italy; 2 CNR-NANOTEC Institute of Nanotechnology, National Research Council, Rende, CS Italy; 3 Department of Chemistry and Chemical Technologies, University of Calabria, Rende, CS Italy; 4 Department of Chemistry, Biology and Biotechnology, University of Perugia, Perugia, Italy; 5 Institute of Light and Matter, UMR 5306, Claude Bernard University Lyon 1, Villeurbanne, France; 6 Institutional Biobank-Scientifc Directorate, National Cancer Institute INT-IRCCS Fondazione G. Pascale, 80131 Naples, Italy; 7 Pathology Unit, National Cancer Institute INT-IRCCS Fondazione G. Pascale, 80131 Naples, Italy; 8 Institute of Biostructures and Bioimaging, National Research Council, 80145 Naples, Italy

**Correction** **:** **J Exp Clin Cancer Res 43, 92 (2024)**

**https://doi.org/10.1186/s13046-024-03014-x**

Following publication of the original article [1], the authors identified an error Figure 7A, middle panel immunohistochemical EGFR staining. The incorrect image for M41 case was included due to mislabeling.

**Incorrect Figure** 7

**Fig. 7:**
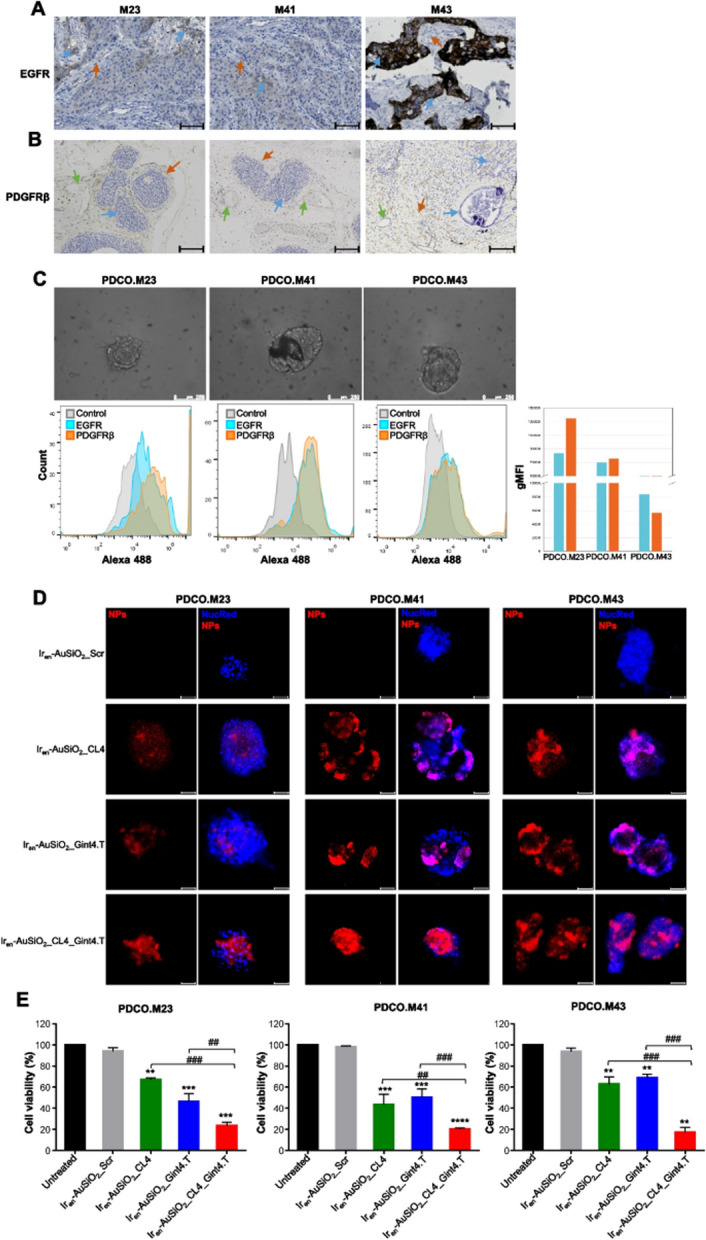
Anticancer activity of Ir_en_-AuSiO_2__Aptamer nanoplatforms on 3D patient-derived breast cancer organoids. Representative images of three breast cancer samples (M23, M41 and M43) stained for **A** EGFR and **B** PDGFRβ. In **A**, the blue arrows indicate EGFR-positive neoplastic cells (M23, moderate membrane expression; M41, mild membrane expression; M43, strong membrane expression); the red arrows indicate EGFR-negative peritumoral stromal cells. Magnifcation: 10 × , scale bar = 100 μm. In **B**, the blue arrows indicate PDGFRβ-negative neoplastic cells; the orange arrows indicate PDGFRβ-positive peritumoral stromal cells (M23 and M41, mild cytoplasmic expression; M43, moderate cytoplasmic expression); the green arrows indicate the endothelial cells of vessels (red blood cells are visible inside) positive for PDGFRβ. Magnifcation: 5 × , scale bar = 50 μm. **C** (upper) Representative phase-contrast microscopy images of PDCOs obtained by M23, M41 and M43 tumor samples, magnifcation: 20 × , scale bar = 250 μm; (lower) fow cytometry analyses to confrm the expression of EGFR and PDGFRβ in the three PDCOs. The histogram indicates the geometric mean fuorescence intensity (gMFI) of EGFR and PDGFRβ expressed on PDCOs, calculated using FlowJo software. **D** Representative confocal images of PDCO.M23, PDCO.M41 and PDCO.M43 incubated with Iren-AuSiO2_CL4, Ir_en_-AuSiO_2__Gint4.T, Ir_en_-AuSiO_2__CL4_Gint4.T or untargeted Iren-AuSiO2_Scr for 24 h at 37 °C. Nanoparticles and nuclei are displayed in red and blue, respectively. Magnifcation: 10 × , 2.0 × digital zoom, scale bar = 50 μm. All digital images were captured under the same settings to enable a direct comparison of staining patterns. **E** Cell viability assay on PDCO.M23, PDCO.M41 and PDCO.M43 treated as indicated. Bars depict mean ± SD of two independent experiments performed in triplicate. **p < 0.01, ***p,0.001, ****p < 0.0001 relative to Ir_en_-AuSiO_2__Scr; ##*p* < 0.01, ###*p* < 0.001, ####*p* < 0.0001. No statistically significant variations among Ir_en_-AuSiO_2__Scr and untreated were obtained

**Correct Figure** 7

**Fig. 7:**
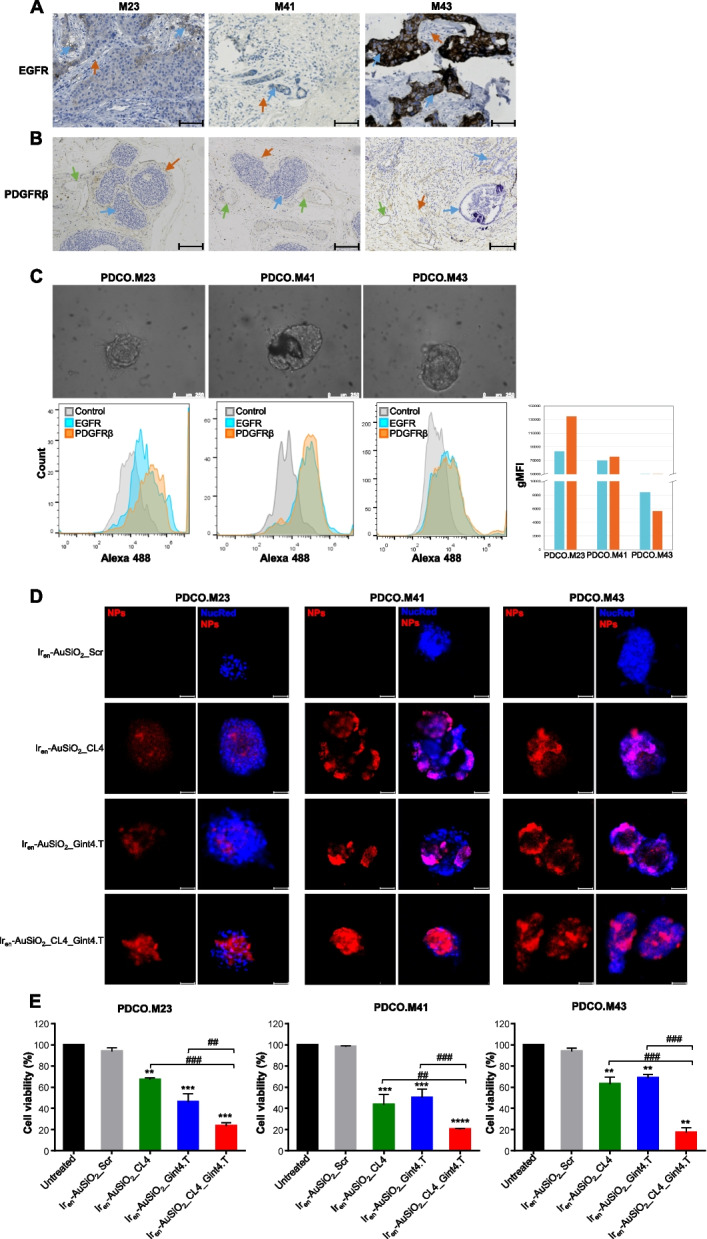
Anticancer activity of Ir_en_-AuSiO_2__Aptamer nanoplatforms on 3D patient-derived breast cancer organoids. Representative images of three breast cancer samples (M23, M41 and M43) stained for **A** EGFR and **B** PDGFRβ. In **A**, the blue arrows indicate EGFR-positive neoplastic cells (M23, moderate membrane expression; M41, mild membrane expression; M43, strong membrane expression); the red arrows indicate EGFR-negative peritumoral stromal cells. Magnifcation: 10 × , scale bar = 100 μm. In **B**, the blue arrows indicate PDGFRβ-negative neoplastic cells; the orange arrows indicate PDGFRβ-positive peritumoral stromal cells (M23 and M41, mild cytoplasmic expression; M43, moderate cytoplasmic expression); the green arrows indicate the endothelial cells of vessels (red blood cells are visible inside) positive for PDGFRβ. Magnifcation: 5 × , scale bar = 50 μm. **C** (upper) Representative phase-contrast microscopy images of PDCOs obtained by M23, M41 and M43 tumor samples, magnifcation: 20 × , scale bar = 250 μm; (lower) fow cytometry analyses to confrm the expression of EGFR and PDGFRβ in the three PDCOs. The histogram indicates the geometric mean fuorescence intensity (gMFI) of EGFR and PDGFRβ expressed on PDCOs, calculated using FlowJo software. **D** Representative confocal images of PDCO.M23, PDCO.M41 and PDCO.M43 incubated with Iren-AuSiO2_CL4, Ir_en_-AuSiO_2__Gint4.T, Ir_en_-AuSiO_2__CL4_Gint4.T or untargeted Iren-AuSiO2_Scr for 24 h at 37 °C. Nanoparticles and nuclei are displayed in red and blue, respectively. Magnifcation: 10 × , 2.0 × digital zoom, scale bar = 50 μm. All digital images were captured under the same settings to enable a direct comparison of staining patterns. **E** Cell viability assay on PDCO.M23, PDCO.M41 and PDCO.M43 treated as indicated. Bars depict mean ± SD of two independent experiments performed in triplicate. **p < 0.01, ***p,0.001, ****p < 0.0001 relative to Ir_en_-AuSiO_2__Scr; ##*p* < 0.01, ###*p* < 0.001, ####*p* < 0.0001. No statistically significant variations among Ir_en_-AuSiO_2__Scr and untreated were obtained

The original article [1] has been corrected.
