# Supplementary material for: Bispecific aptamer-decorated and light-triggered nanoparticles targeting tumor and stromal cells in breast cancer derived organoids: implications for precision phototherapies
Source: J Exp Clin Cancer Res. 2024 Mar 26;43:92. doi: 10.1186/s13046-024-03014-x (PMC10964525; doi:10.1186/s13046-024-03014-x)
Supplement: Supplementary file 1 — Additional file 1: Supplementary Figure 1. Characterization of Iren-AuSiO2_COOH nanoparticles. Supplementary Figure 2. Light-induced singlet oxygen generation of Iren-AuSiO2_COOH. Supplementary Figure 3. Light-induced photothermal effect of Iren-AuSiO2_COOH. Supplementary Figure 4. Extinction spectra acquired in the time range 0-28 days of aptamers-conjugated nanoparticles dispersed in water. Supplementary Table 1. Properties of aptamers-nanoplatforms conjugates. Supplementary Figure 5. Quantification of the amount of aptamer conjugated to Iren-AuSiO2_COOH/NHS. Supplementary Figure 6. Expression of PDGFRβ and EGFR in different human cell lines. Supplementary Figure 7. Selective cell uptake of CL4 and/or Gint4.T-decorated Iren-AuSiO2_Aptamer nanoplatforms in 2D BT-549 or BT-474 cultures. Supplementary Figure 8. Formation of 3D spheroids of BT-549 cells and MSC. Supplementary Figure 9. Photodynamic effect of nanoplatforms in 2D cell cultures. Supplementary Figure 10. Formation of 3D spheroids of EGFR+/PDGFRβ− cancer cells and MSC. Supplementary Table 2. Clinicopathological features of three selected tumor samples. [file 13046_2024_3014_MOESM1_ESM.pdf]

Supplementary Information

Bispecific aptamer-decorated and light-triggered nanoparticles targeting tumor and stromal cells in breast cancer derived organoids: implications for precision phototherapies

Simona Camorani[1], Alessandra Caliendo[1], Elena Morrone[2,3,4], Lisa Agnello[1], Matteo Martini[5], Monica Cantile[6], Margherita Cerrone[7], Antonella Zanetti[8], Massimo La Deda[2,3], Monica Fedele[1], Loredana Ricciardi[2*], Laura Cerchia[1*]

[1]Institute of Experimental Endocrinology and Oncology "Gaetano Salvatore", National Research Council, 80131, Naples, Italy.

[2]CNR-NANOTEC Institute of Nanotechnology, National Research Council, Rende (CS), Italy.

[3]Department of Chemistry and Chemical Technologies, University of Calabria, Rende (CS), Italy.

[4]Department of Chemistry, Biology and Biotechnology, University of Perugia, Italy.

[5]Institute of Light and Matter, Claude Bernard University Lyon 1, UMR 5306, Villeurbanne, France.

[6]Institutional Biobank-Scientific Directorate, National Cancer Institute INT-IRCCS Fondazione G. Pascale, 80131, Naples, Italy.

[7]Pathology Unit, National Cancer Institute INT-IRCCS Fondazione G. Pascale, 80131, Naples, Italy.

[8]Institute of Biostructures and Bioimaging, National Research Council, 80145, Naples, Italy.

[*]Correspondence: l.cerchia@ieos.cnr.it; loredana.ricciardi@cnr.it

A

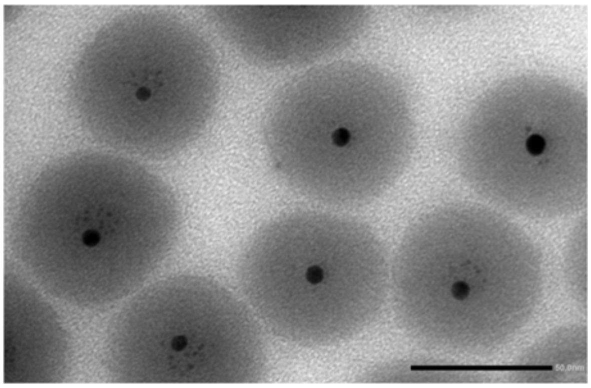

B

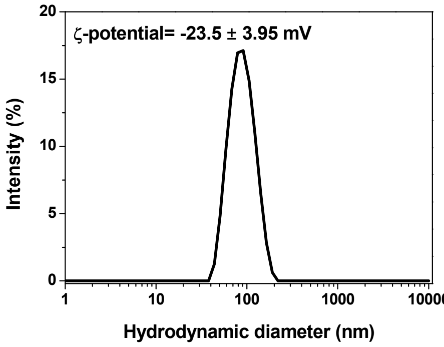

C

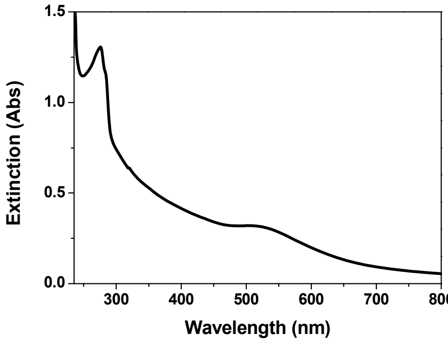

D

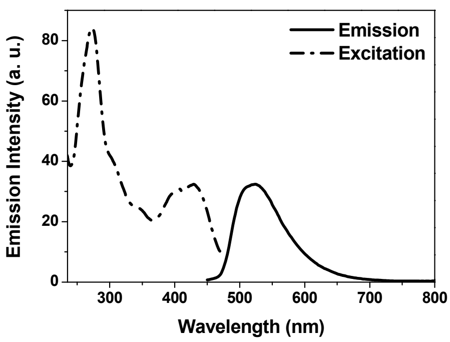

Supplementary Figure 1. Characterization of Ir_{en}-$AuSiO_2$_COOH nanoparticles. **A** Representative TEM image. Scale bar: 50 nm. **B** Hydrodynamic diameter and ζ-potential value, **C** extinction spectrum. **D** excitation and emission spectra (λ_{ex}=420 nm; λ_{em}=520 nm) of Ir_{en}-$AuSiO_2$_COOH dispersed in water.

Light-induced singlet oxygen generation and photothermal effects of Ir_{en}-$AuSiO_2$_COOH

The generation of singlet oxygen by the photosensitizer-doped gold-silica nanoparticles was validated by monitoring spectrophotometrically the chemical oxidation of the molecular probe 9,10-Anthracenediyl-bis(methylene)dimalonic acid (ABDA) (Sigma-Aldrich, St. Louis, MO, USA) in

aqueous solution. The ABDA molecule reacts irreversibly with singlet oxygen to form the corresponding endoperoxide, resulting in attenuation of the absorption peak at 378 nm.

Briefly, 15 µL of ABDA solution (2 mg/mL DMSO) were mixed with 0.75 mL of Ir_{en}-$AuSiO_2$_COOH solution and placed in a quartz cuvette with an optical path length of 0.2 cm. The generation of singlet oxygen was monitored upon irradiation at 254 nm for 15 min (five cycles of 3 min) by a xenon discharge lamp (equivalent to 20 kW for 8 µs duration). As control solution, 15 µL of ABDA solution were mixed with 0.75 mL of water and irradiated under the same conditions.

A

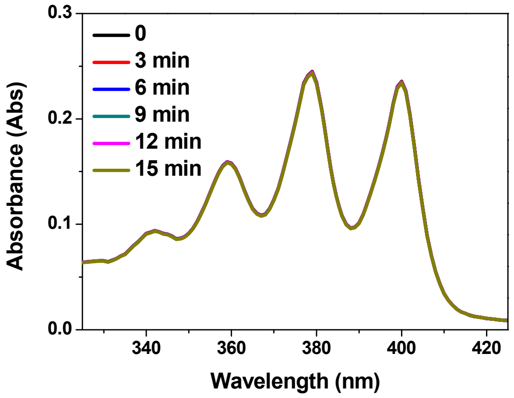

B

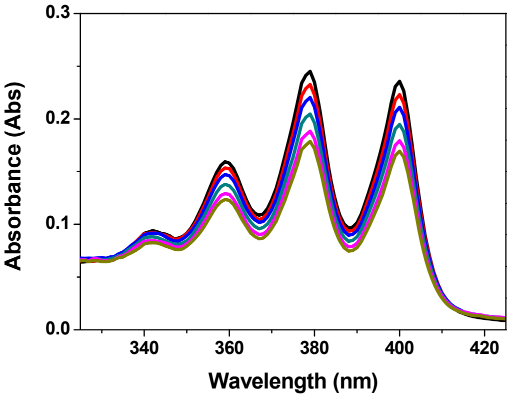

C

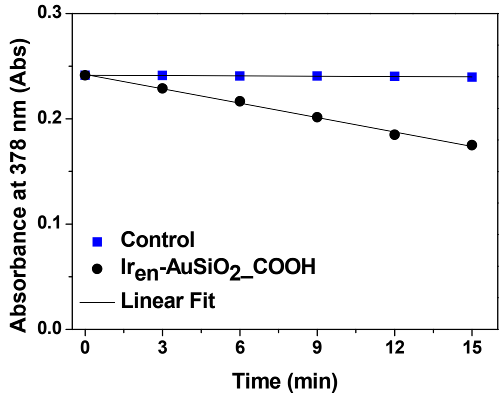

Supplementary Figure 2. Absorbance attenuation of ABDA by singlet oxygen generation. **A** Photobleaching of ABDA at different irradiation times in water (control) and **B** in Ir_{en}-

$AuSiO_2$_COOH aqueous solution. **C** Plotting of ABDA absorption at 378 nm as a function of illumination time.

The nanoplatforms heat generation under continuous illumination

To validate the photothermal effects, the thermal behavior of Ir_{en}-$AuSiO_2$_COOH nanoparticles was investigated under a 450 W continuous Xenon lamp irradiation; a photograph of the experimental setup used to carry out the measurements is shown in Supplementary Figure 3A. The incident light beam (254 nm) impinged on a 1 x 0.4 cm quartz cuvette containing 1 mL of nanoparticles solution. A thermal camera (C-3X model, by FLIR), characterized by a sensitivity of 0.07°C and placed perpendicular to the incident light (forming a 90-degree angle with the surface of the cuvette), was used to monitor the photo-induced temperature variations. The chamber temperature before the start of irradiation, for both control solution (water) and sample, was 22.1°C. After 90 min of irradiation, a thermal image, showing the value of the minimum and maximum temperature recorded in the area of interest, was acquired (Supplementary Fig. 3B,C).

A **B** **C**

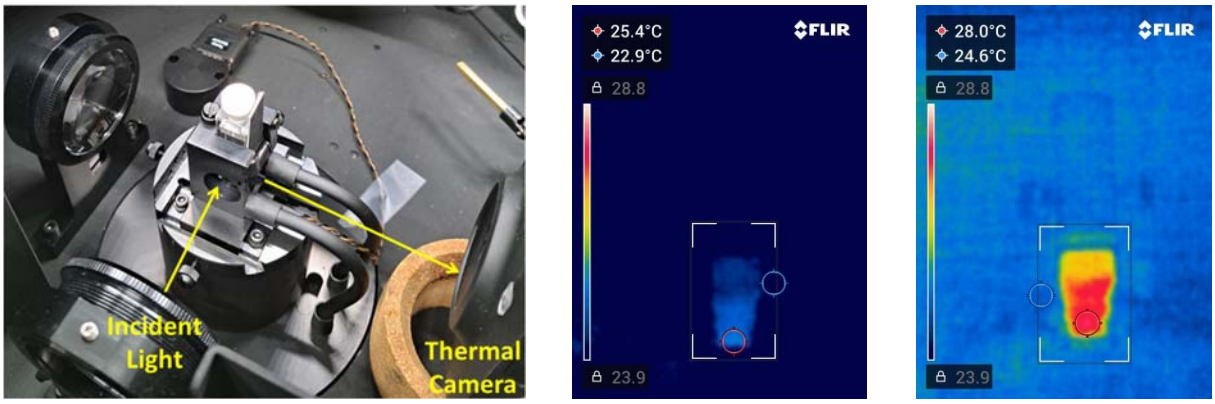

Supplementary Figure 3. Light-induced photothermal effect of Ir_{en}-$AuSiO_2$_COOH. **A** Picture of the used measurement setup to investigate the light-induced thermal effect of Ir_{en}-$AuSiO_2$_COOH. Infrared thermal images of **B** water (control) and **C** Ir_{en}-$AuSiO_2$_COOH nanoparticles dispersed in water (light exposure 254 nm, 90 min).

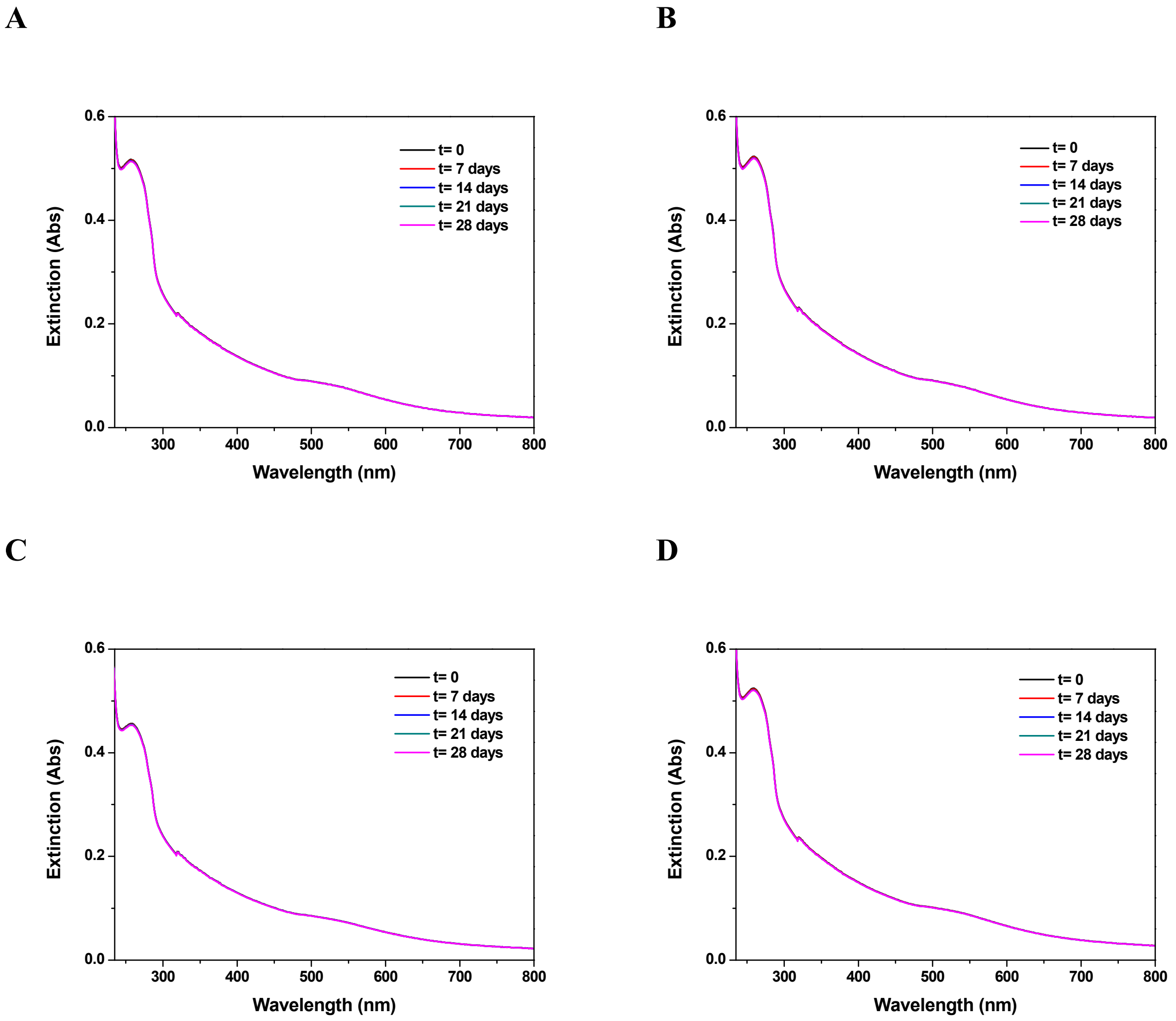

Supplementary Figure 4. Extinction spectra acquired in the time range 0-28 days of aptamers-conjugated nanoparticles dispersed in water. Extinction spectra of **A** Ir_{en}-$AuSiO_2$_CL4, **B** Ir_{en}-$AuSiO_2$_Scr, **C** Ir_{en}-$AuSiO_2$_Gint4.T and **D** Ir_{en}-$AuSiO_2$_CL4_Gint4.T dispersed in water over 7, 14, 21 and 28 days.

Supplementary Table 1. Properties of aptamers-nanoplatforms conjugates

Nanoparticles formulation	Hydrodynamic diameter (nm)	PDI	ζ-potential (mV)
**Ir_{en}-$AuSiO_2$_COOH/NHS**	102.0 ± 0.15	0.172	-26.0 ± 1.01
**Ir_{en}-$AuSiO_2$_CL4**	102.1 ± 0.93	0.163	-22.3 ± 2.12
**Ir_{en}-$AuSiO_2$_Scr**	101.4 ± 0.20	0.162	-22.9 ± 2.25
**Ir_{en}-$AuSiO_2$_Gint4.T**	104.4 ± 0.47	0.168	-28.3 ± 3.12
**Ir_{en}-$AuSiO_2$_CL4_Gint4.T**	103.7 ± 0.82	0.162	-26.4 ± 8.12

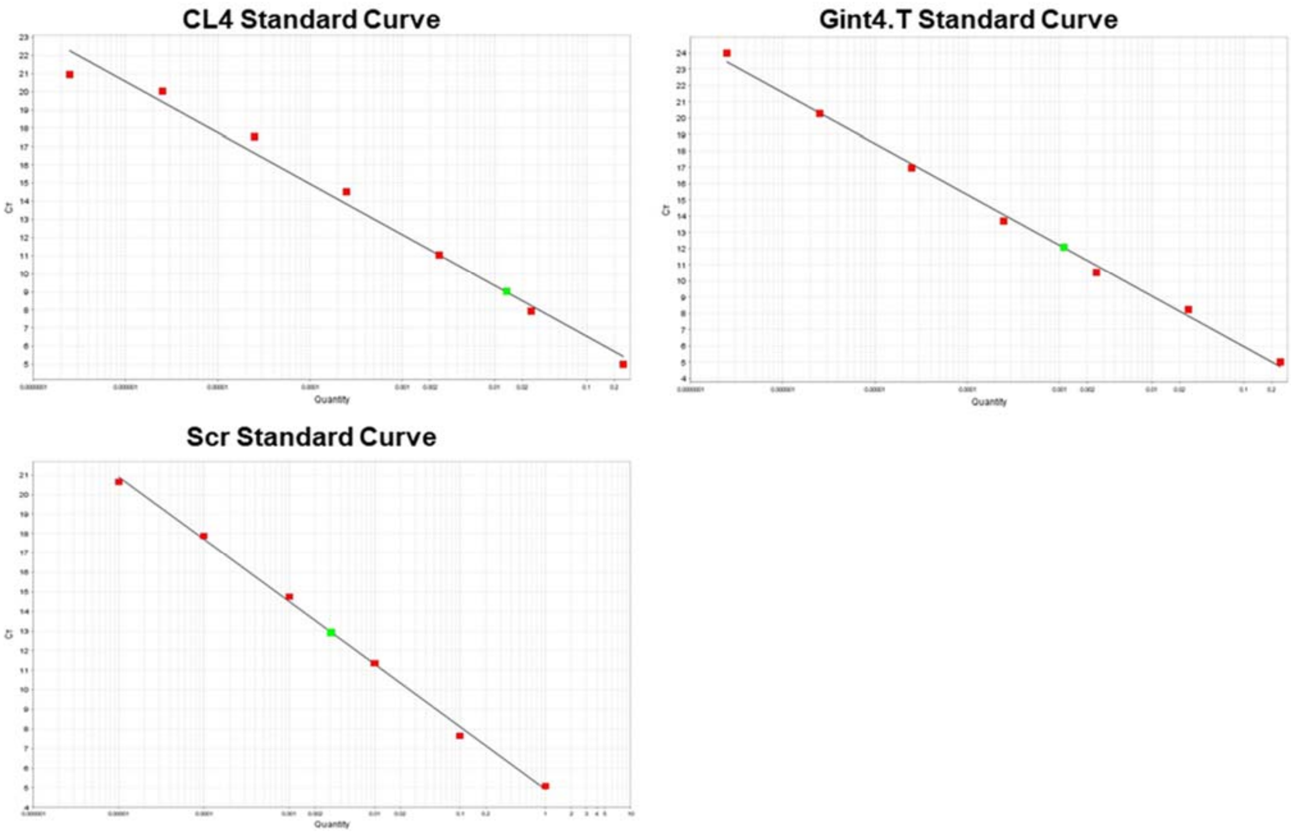

Supplementary Figure 5. Quantification of the amount of aptamer conjugated to Ir_{en}-$AuSiO_2$_COOH/NHS. PCR calibration curve with unconjugated aptamer (red); sample, green.

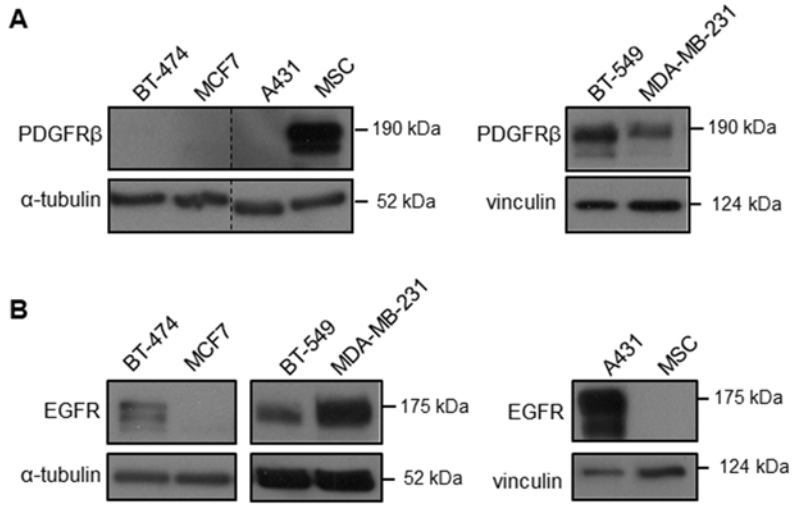

Supplementary Figure 6. Expression of PDGFRβ and EGFR in different human cell lines. Lysates from cancer BT-474, MCF7, MDA-MB-231, BT-549, and A431 cell lines and stromal MSCs were immunoblotted with **A** anti-PDGFRβ or **B** anti-EGFR antibodies, as indicated. Equal loading was confirmed by immunoblot with anti-vinculin or anti-α-tubulin antibody, as indicated. The molecular weights of the indicated proteins are reported. In **A**, black dashed lines delineate the boundary between non-contiguous lanes of the same gel.

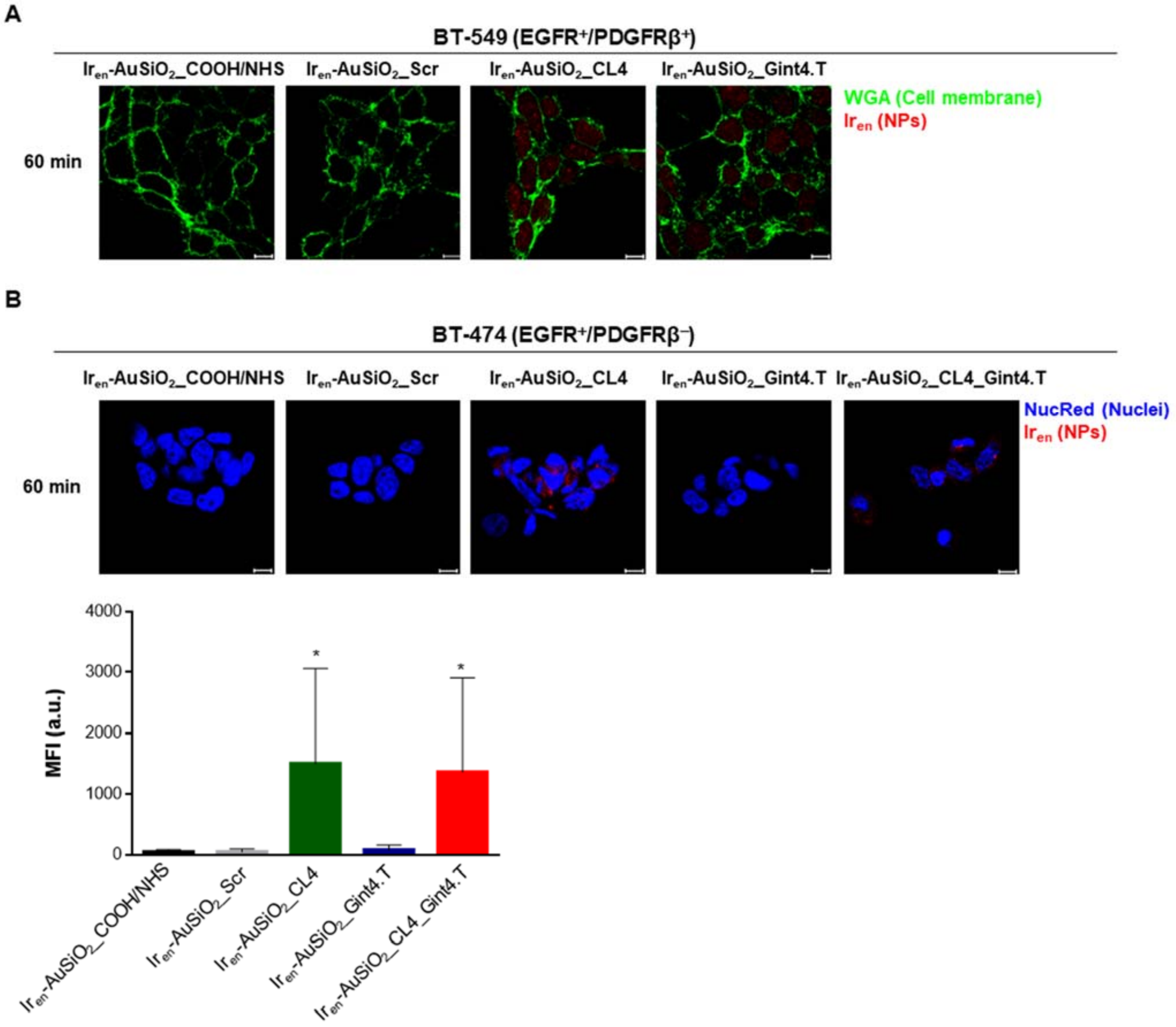

Supplementary Figure 7. Selective cell uptake of CL4 and/or Gint4.T-decorated Ir_{en}-$AuSiO_2$_Aptamer nanoplatforms in 2D BT-549 or BT-474 cultures. **A** Representative confocal images of BT-549 ($EGFR^+/PDGFR\beta^+$) cells incubated for 60 min at 37°C with Ir_{en}-$AuSiO_2$_COOH/NHS or Ir_{en}-$AuSiO_2$_Aptamer nanoparticles. After washing and fixation, cells were labeled with WGA-488 (green) to visualize the cell membrane. **B** Representative confocal images of BT-474 ($EGFR^+/PDGFR\beta^-$) cells incubated for 60 min at 37°C with the indicated nanoparticles. After washing and fixation, cells were labeled with NucRed (blue) to stain nuclei. Mean fluorescence intensity (MFI) was evaluated by Zeiss software on 10 separate images for each condition. Bars depict means ± SD (n = 3). * $p < 0.05$ relative to Ir_{en}-$AuSiO_2$_Scr. **A**,**B** Nanoparticles are displayed in red. All digital images were captured at the same

setting to allow direct comparison of staining patterns. Magnification 63×, 1.0× digital zoom, scale bar = 10 µm.

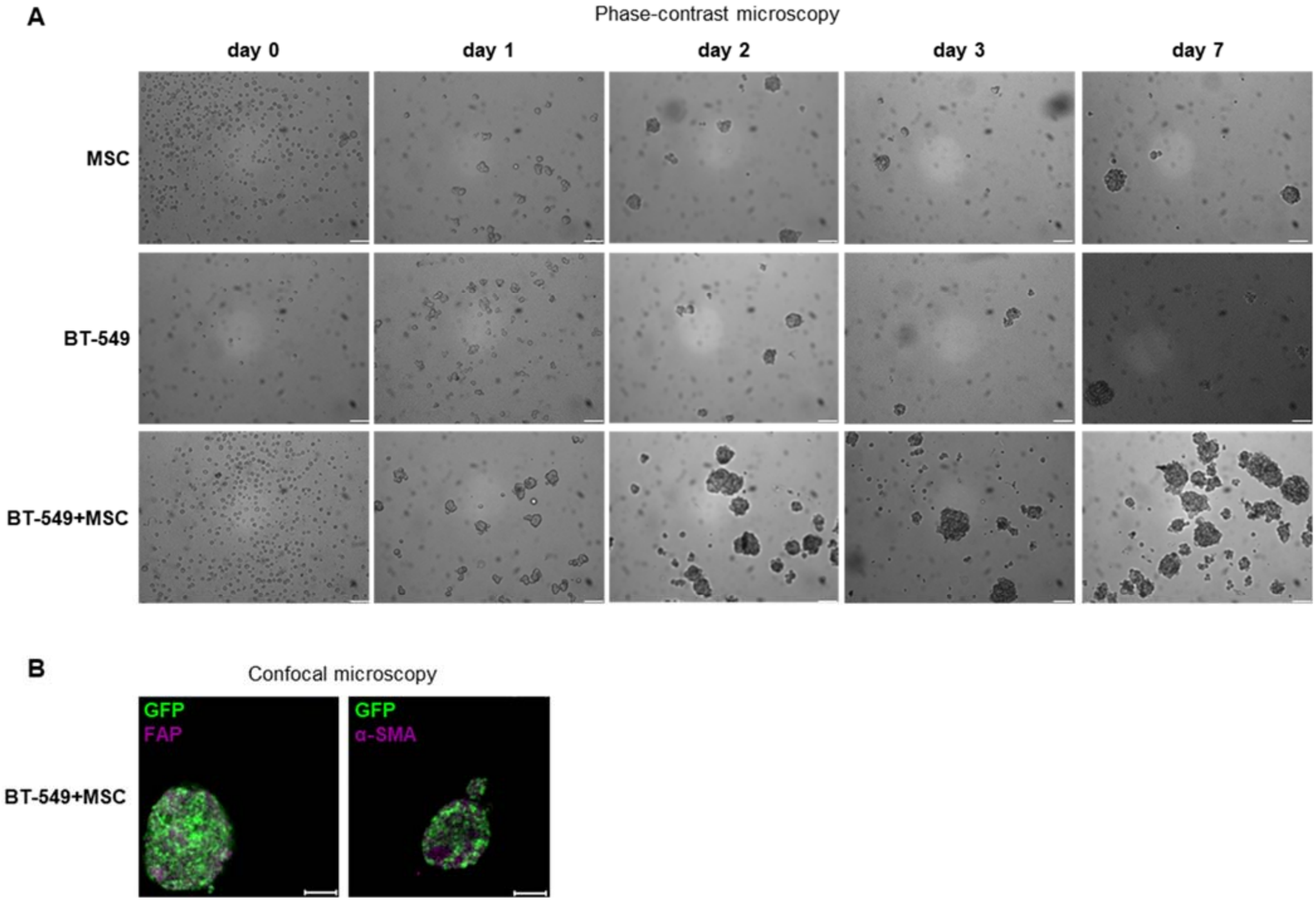

Supplementary Figure 8. Formation of 3D spheroids of BT-549 cells and MSC. **A** Representative phase-contrast microscopy images showing the formation of homotypic (MSC; BT-549) and heterotypic (BT-549+MSC) spheroids over seven days. Magnification: 10×, scale bar = 100 µm. **B** Representative confocal images of the heterotypic spheroids grown at day 13 and then stained with α-SMA and FAP stromal markers. BT-549-GFP cells and stromal markers are displayed in green and magenta, respectively. Magnification: 10×, 1.0× digital zoom, scale bar = 100 µm.

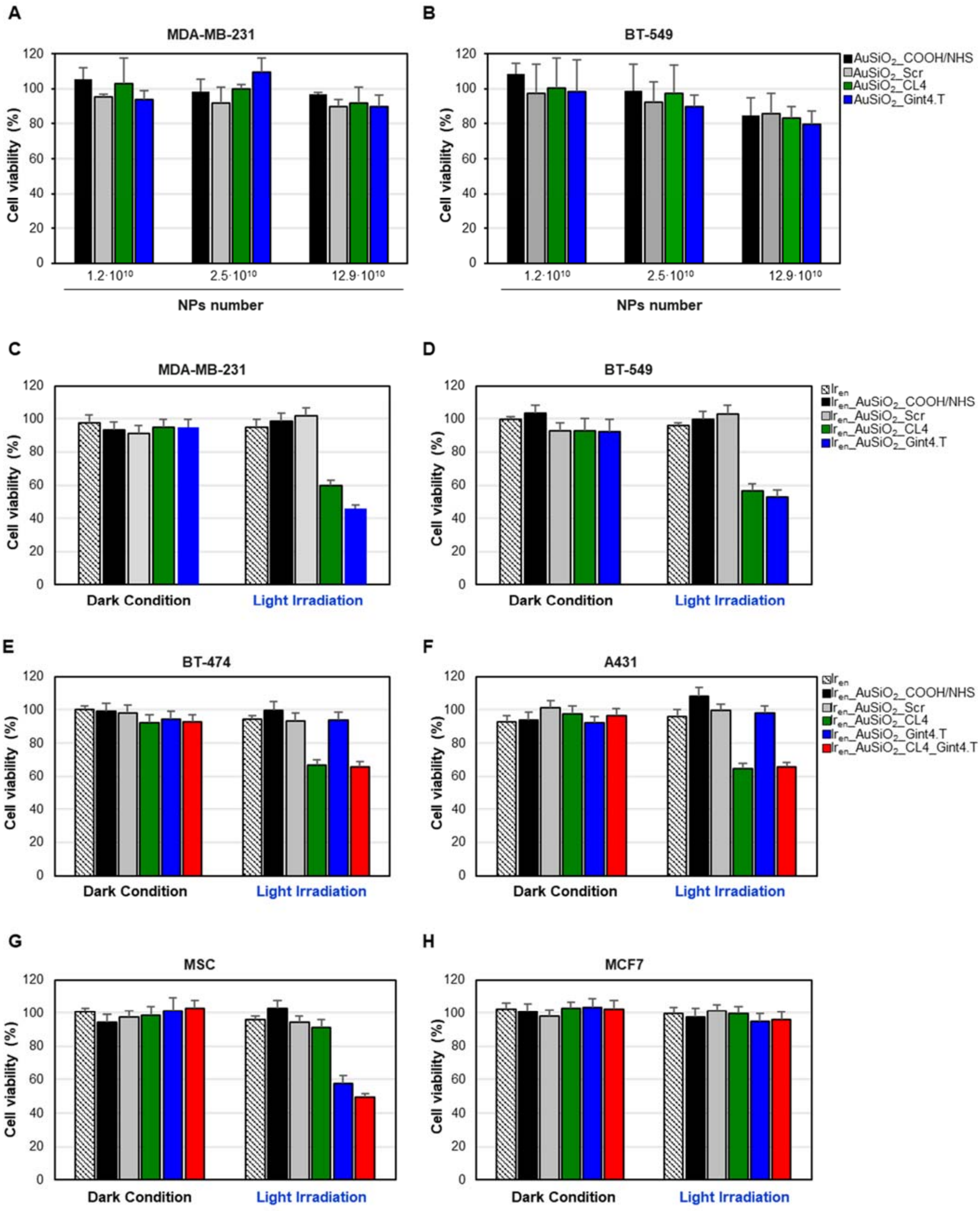

Supplementary Figure 9. Photodynamic effect of nanoplatforms in 2D cell cultures. **A**,**B**, AuSiO2_COOH/NHS, AuSiO2_Scr, AuSiO2_CL4 or AuSiO2_Gint4.T, without the photosensitizing

and luminescent molecule Ir_{en}, were incubated onto MDA-MB-231 or BT-549 cells for 24 h and cell viability was assessed to exclude basal toxicity. **C**,**D** MDA-MB-231 and BT-549 ($EGFR^{+}/PDGFR\beta^{+}$) cells, **E**,**F** BT-474 and A431 ($EGFR^{+}/PDGFR\beta^{-}$) cells, **G** MSCs ($EGFR^{-}/PDGFR\beta^{+}$), and **H** MCF7 ($EGFR^{-}/PDGFR\beta^{-}$) cells, were treated with free Ir_{en}, Ir_{en}-$AuSiO_2$_Aptamer or unconjugated Ir_{en}-$AuSiO_2$_COOH/NHS nanoparticles for 60 min at 37°C, washed and kept in the dark or exposed to 254 nm light irradiation for 1 h. After 24 h, cell viability was assessed. Treatment with specific aptamer-decorated nanoplatforms, but not with free Ir_{en}, Ir_{en}-$AuSiO_2$_COOH/NHS or Ir_{en}-$AuSiO_2$_Scr, inhibits cell viability, expressed as percentage of viable treated cells with respect to untreated cells. **A**-**H** Bars depict mean ± SD (n = 3). No statistically significant variations among Ir_{en}-$AuSiO_2$_COOH/NHS or Ir_{en}-$AuSiO_2$_Scr and untreated were obtained.

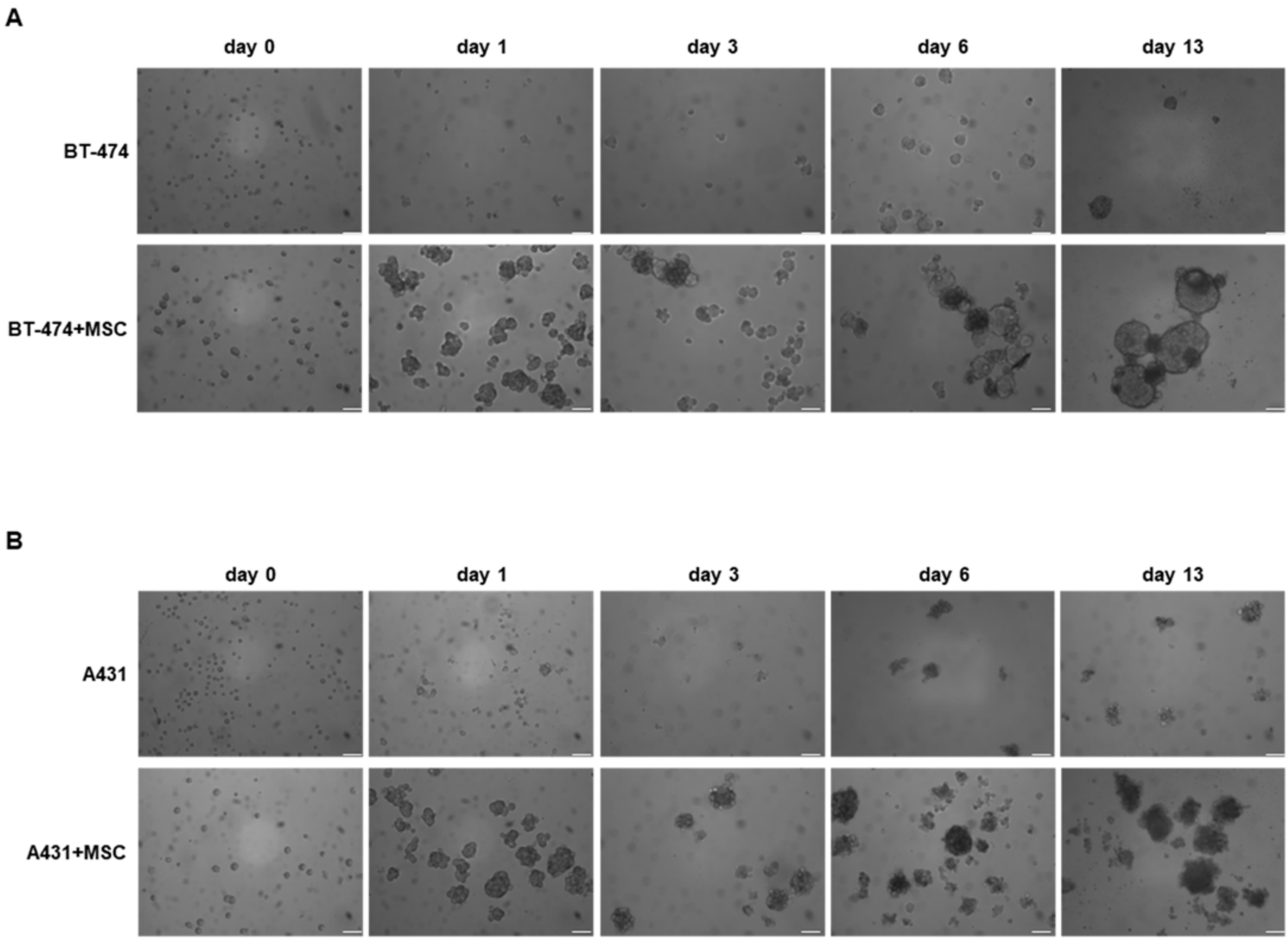

Supplementary Figure 10. Formation of 3D spheroids of EGFR^{+}/PDGFRβ^{-} cancer cells and MSC. **A**,**B** Representative phase-contrast microscopy images showing the formation of homotypic spheroids of BT-474 or A431 cells, and heterotypic spheroids of BT-474 or A431+MSCs over thirteen days. Magnification: 10×, scale bar = 100 μm.

Supplementary Table 2. Clinicopathological features of three selected tumor samples.

Tumor sample	Age	Histological diagnosis	TNM stage	ER	PgR	HER2	Ki67
M23	63	Ductal carcinoma in situ (DCIS)	pTis pN0	90%	80%	NA	10%
M41	47	Invasive carcinoma no special type (NST)	pT2G 2N2a	90%	90%	Score 1+	28%
M43	71	Invasive carcinoma no special type (NST)	pT2G 3Nx	90%	80%	Score 1+	40%
